# Assessing psychological and physical abuse from children’s perspective: Factor structure and psychometric properties of the picture-based, modularized child-report version of the Parent-Child Conflict Tactics Scale – Revised (CTSPC-R)

**DOI:** 10.1371/journal.pone.0205401

**Published:** 2018-10-08

**Authors:** Susan Sierau, Lars Otto White, Annette Maria Klein, Jody Todd Manly, Kai von Klitzing, Philipp Yorck Herzberg

**Affiliations:** 1 Department of Child and Adolescent Psychiatry, Psychotherapy and Psychosomatics, University of Leipzig, Leipzig, Germany; 2 Department of Medical Psychology and Medical Sociology, University of Leipzig, Leipzig, Germany; 3 International Psychoanalytic University Berlin, Berlin, Germany; 4 Mount Hope Family Center, University of Rochester, Rochester, New York, United States of America; 5 Helmut Schmidt University, University of the Federal Armed Forces Hamburg, Hamburg, Germany; Harvard Medical School, UNITED STATES

## Abstract

Child victims’ reports of psychological and physical abuse by caregivers are a fundamental source of information beyond official records and caregiver reports. However, few or no sensitive and age-appropriate child-report instruments exist that have undergone in-depth validity and reliability testing across a broad age-range. Our study addresses this gap by examining psychometric properties of a picture-based, modularized version of the Parent-Child Conflict Tactics Scale (CTSPC-R), encompassing the maltreatment subtypes of psychological and physical abuse. A sample of 904 children and adolescents aged 4–16 years from the community (n = 568), child psychiatric services (n = 159), and from Child Protective Services (CPS; n = 177) completed the CTSPC-R. Measures to test convergent (maltreatment in parent interviews and CPS records) and concurrent validity (psychiatric symptoms) were collected. The CTSPC-R comprises 22 items, arranged in three severity modules by increasing level of psychological and physical abuse by caregivers. Companion picture cards were provided for children aged 4 and 8 years. The best fit to the data was attained with a second-order factor model, assuming three inter-correlated factors corresponding to the three severity modules, and a latent second-order factor representing combined physical and psychological abuse. The three factors showed good internal consistencies. Supporting convergent validity at the global and subtype-level of maltreatment, the CTSPC-R severity scale was associated with lifetime CPS-contact, presence of caregiver-reported emotional maltreatment and physical abuse, and dimensions of chronicity and severity. Discriminant validity was supported by non-significant correlations with caregiver-reported lack of supervision, failure to provide, and sexual abuse. Bolstering concurrent validity, moderate and severe physical abuse predicted caregiver-reported internalizing and externalizing symptoms. These effects were independent of child age, gender or community vs. non-community samples. Our study supports the CTSPC-R as a scientifically and clinically sound tool for ascertaining the child’s own perspective on psychological and physical abuse from an early age onwards.

## Introduction

Childhood exposure to psychological and physical abuse represents a major risk for poor physical and mental health throughout the life course [1–4]. Yet despite UNICEF’s call for the “child’s right to be heard” in cases of violence [5], very few, if any, instruments currently exist that have proven valid and reliable in sampling the child’s perspective on maltreatment. This study aimed to fill this crucial gap by adapting an existing instrument for use across a wider age-range from preschool-age to adolescence as well as across low-risk community and high-risk child psychiatric/CPS settings.

The development and application of reliable and valid self-report measurement scales on physical violence follows a long tradition, beginning with the Conflict Tactics Scales (CTS) [6,7] as one of the most prominent and widely used maltreatment instruments in epidemiological and clinical studies (for an overview of CTS adaptations and their psychometric evaluation see [8]). The theoretical basis for this measure is conflict theory [7], which assumes that while conflict is inherent to all human relationships, physical assault is a maladaptive, dysfunctional, and destructive strategy.

The CTS, originally developed to assess violence in intimate relationships, was revised and adapted for the use in parent-child relationships [9]. The Parent-Child Conflict Tactics Scales (CTSPC) [10] were validated as a parent-report screening tool for epidemiological and clinical assessments capturing negative parental conflict tactics or acts of physical and emotional violence towards the child in the previous year. The CTS and the CTSPC are based on the same theoretical and measurement strategies that allow estimation of prevalence rates of psychological and physical maltreatment [9]. The psychometric properties of the CTSPC parent-report have been tested in a representative sample of 1,000 U.S. households with children under 18 years of age via telephone interviews with parents [10]. More recently, studies on the CTSPC child-report found evidence for its validity with significant associations between child-report of abusive parenting and child externalizing symptoms in 8 to 10-year-olds [11,12], and delinquency in 12 to 19-year-olds [13] as well as symptoms of Borderline Personality Disorder in 14-year-old girls [14]. Furthermore, child-report on harsh punishment and abusive parenting was especially in boys related to parasympathetic activity [12,15], and sleep behavior [16] as a moderator of developmental risk for behavioral problems in childhood and adolescence. However, these studies either used combined scores of child-reports on psychological and physical maltreatment (called ´harsh punishment´ or ´abusive parenting´) [11,12,15,16] or a brief scale including a limited number of CTSPC items [14]. Thus, the question remains if the factor structure of the CTSPC parent-report also applies to children, and if these factors are reliable and valid. Furthermore, there is a paucity of data on child reports establishing reliability (e.g., internal consistency, test-retest reliability) and validity, especially convergent and discriminant validity.

The child’s perspective on maltreatment incidences can be a key element in the decision making-processes of the Child Protection Services (CPS) but is also useful in the detection of cases which are not known to the CPS [17]. Several issues must be considered when assessing child maltreatment via self-reports of children. First, the age or the developmental level of the child may influence item comprehension and, therefore, the consistency of responses. In particular, extended referent periods or highly severe or traumatic events may be difficult to recall for a child and can decrease accuracy of reports. Cues such as pictures can scaffold recollection of those experiences [18]. Second, reporting biases may result in distortion. Specifically, children may be especially reluctant to report negative behavior of their parents (e.g. due to feelings of loyalty), and therefore, their reports may be biased by their feelings of shame, fear or guilt. A face-to-face interview situation in a private atmosphere may be most conducive to a valid report by children. Moreover, beyond the promise of confidentiality, a matter-of-fact explanation implying that things to be asked about can and do happen to children may help normalize children’s experience and thus create a climate of openness and trust [10]. Third, the threshold for labelling an incidence as maltreatment is often unclear and not easily detectable for researchers and clinicians. The CTSPC takes normative factors into consideration by distinguishing between practices of nonviolent discipline, psychological aggression, and physical abuse [9]. In addition, external criteria can help to evaluate the classification performance of instruments.

Given these challenges of assessing maltreatment experiences, most researchers are reluctant to conduct interviews on maltreatment, and particularly on severe maltreatment incidences, with young children below age 8. Of the little research in this area, DuMont et al. [19] assessed CTSPC nonviolent discipline, psychological aggression, and minor physical assault in 7-year-olds whose families took part in a home-visitation program. Yet due to concerns of the authors about exposing children to more severe pictures, incidences of severe physical assault were not considered, although documenting these incidences is crucial for adequate treatment.

To enable the age-appropriate and sensitive assessment of psychological and physical abuse in preschool-age children from age 4 onwards, we adapted the CTSPC by developing a picture-based, modularized version, the CTSPC-R. Importantly, to minimize exposure of children to potentially distressing item content as best as possible, we created arranged modules in a three-step sequence of increasing severity of item content. The first module (minor severity) represents a “core item set” which was administered to all children and contains instances of nonviolent discipline, psychological aggression, and corporal punishment. The second module (moderate severity) includes incidences of severe corporal punishment and physical abuse. The third module (high severity) contains items of severe physical abuse. Modules 2 and 3 were only administered if children endorsed a specific kind or number of items from the core item set (for further details of the CTSPC-R, see below).

The present study analyzes the distributional and psychometric properties of the CTSPC-R in a large, heterogeneous sample of community and CPS-involved families with 4- to 16-year-olds. We examined the factor structure of the CTSPC-R by using confirmatory factor analysis for comparison of four different models as well as model modification and cross-validation. In addition, we tested for construct (i.e. convergent and discriminant), and criterion-related (i.e. concurrent) validity as well as for influences of risk-setting, child age and gender to verify the consistency as well as the accuracy of the factors. Our overarching aim is to provide a self-report measure on physical and psychological abuse for children from early childhood to adolescence, to inform our understanding of young children’s perception of these incidences, with the eventual possibility of integration into multi-informant research on maltreatment and applied CPS and child psychiatric contexts.

## Methods

### Participants

Study participants were 904 children and adolescents aged 4–16 years (age: mean = 10.3, SD = 3.1 years; n = 345 [38.2%] 4 to 8 years; n = 559 [61.8%] 9 to 16 years), and their primary caregiver (91.8% biological mother, 7.4% another female relative or mother figure, 0.8% biological father) from three German samples (s1-s3) with different risk-settings who participated in the AMIS project (Analyzing pathways from childhood maltreatment to internalizing symptoms and disorders in children and adolescents; see White et al. [20] for a detailed description of the project). The first sample (s1) included 568 children and adolescents (age: mean = 9.9, SD = 3.1 years; 294 [51.8%] female]) from the community. Within this sample, 14.1% (n = 74) had, at one point or another, received services from the Youth Welfare Office, and 4.2% (n = 21) were reported to the CPS at least once in their lifetime. The second sample (s2) included 159 children and adolescents (age: mean = 12.4, SD = 1.8; 58 [36.5%] female]) who received child psychiatric services. Within this sample, 50.0% (n = 78) had received services from the Youth Welfare Office, and 20.5% (n = 32) were reported to the CPS. The third sample (s3) comprised 177 children and adolescents (age: mean = 9.8 years, SD = 3.2, 82 [46.3%] female]) from CPS with documented maltreatment.

### Procedures

The study has been approved by the respective ethics committees of the Universities of Leipzig and Munich, Germany (registration numbers: 178-12-21052012, 098-12-05032012, and 098-12-05032012). Parents were informed orally and in written form about the contents and aims of the study and gave their written consent in order to enroll. All participation was voluntary and could be withdrawn by the family at any time without penalty or consequences of any kind. All procedures were in accordance with the Helsinki Declaration. Prior to giving consent, participants were informed of the procedures that would be undertaken if recent incidences of maltreatment that were not known to CPS were revealed to the interviewers. Families were reimbursed for their participation in the study. Assessments were conducted in parallel sessions with children and their primary caregivers in separate rooms at the lab to ensure privacy. Trained researchers administered all assessment methods and received regular supervision to ensure high quality of data collection. Clinical supervision was provided by an external supervisor to discuss difficult interview situations. If recent incidences of maltreatment that were not known to CPS were revealed to the interviewers, the research team organized a child protection conference which included the medical director of the department and the principal investigator of the study. As a first step, the parents and the child received consultation and recommendations regarding clinical service. If necessary, the researchers strived to obtain permission of the caregiver to contact the CPS in order to organize support for the family. However, in one case in which the caregivers did not give permission and the situation was so threatening that it required urgent action, CPS was involved even without permission of the caregiver. This procedure was in accordance with the legal obligations in the country.

### Measures

#### The picture-based, modularized parent-child conflict tactics scales (CTSPC-R)

The CTSPC-R comprises 22 items from Straus et al.’s [10] CTSPC capturing acts of non-violent discipline, and psychological and physical abuse towards the child in the previous year. Items are rated on a five-point scale (0 = “did not occur”, 1 = “did occur once”, 2 = “did occur a few times”, 3 = “many times”, 4 = “every time”) [21] separately for the primary and secondary caregiver (usually the parents; perpetrator A and B). In order to create a context in which children feel safe in disclosing maltreatment experiences, a brief explanation was given implying that many children experience the things to be asked about. In addition, a visual presentation of the five-point scale also allowed pointing instead of giving a verbal answer (based on [18]).

We developed age-appropriate picture-versions of all items with the help of a children’s book illustrator (see S1 File for pictures on all CTSPC items). Drawings were presented with each item for all children aged 4 to 8 years to improve the understanding of item content. From 9 years onwards, drawings were only provided if concerns existed regarding item understanding. To interview children in a careful and developmentally sensitive manner, items were presented in a three-step fashion: Module 1 (minor severity, 15 items) comprised incidences of nonviolent discipline and psychological aggression as well as less severe physical maltreatment (e.g., slapping, shaking, pinching). Module 2 (moderate severity, 4 items) covered events of physical abuse with moderate severity (e.g., spanking, hitting, beating up, knocking down) while module 3 (high severity, 3 items) covered highly severe events of physical abuse (e.g., cigarette burns, threatening with a knife or gun, choking). Three items from module 1 covering less severe physical assaults (including items 16 and 7) needed to be endorsed before completing module 2 (i.e., rated 1 or higher), and three items from module 2 (moderate severity) needed to be endorsed before completing module 3 (high severity). A consensus regarding the selection of item sets and cut-off criteria for each module was achieved following multiple consultations with clinical and research experts on child abuse. In line with the CTS, sets and items within sets were presented in a hierarchical sequence from high to low social acceptability [7]. In the study of Straus et al. [10], nonviolent parenting was associated with absence of physical violence, supporting the administration of items in modules based on severity. A score of "0" was assumed for the items from higher sets that were not administered to some participants as the cut-off was not reached (see also [22]). Item descriptions and item means for perpetrator A and B in order of presentation are displayed in Table 1. Children responded each item about both caregivers before moving onto the next item. In the present study, 97.9% (n = 885) reported on at least one caregiver (perpetrator A), and 95.5% (n = 863) of the children also reported on a second caregiver (perpetrator B). A minority of children (2.1%, n = 19) did not complete the CTSPC-R due to denial of further study participation and were excluded from analyses. There were no differences between those included in this analysis and those not included based on child age, gender, or sample characteristics (*p* >.05). Due to the extremely skewed distribution of most items covering rare events, we followed the procedure of Straus et al. [10] and conducted analyses of validity based on prevalence rates instead of mean scores. A total of 91.1% (n = 806) endorsed items in module 1 for perpetrator A, and 81.8% (n = 706) of the children endorsed items in this module for perpetrator B. In module 2, 4.3% (n = 38) of the children endorsed items for perpetrator A, and 3.0% (n = 26) of the children endorsed items in this module for perpetrator B. In module 3, 1.5% (n = 13) of the children endorsed items for perpetrator A, and 1.6% (n = 14) of the children endorsed items in this module for perpetrator B. 7.1% (n = 64) reported no incidences of psychological or physical abuse for any of the two caregivers. Internal consistencies and further psychometric properties of the CTSPC-R are reported in the results section.

**Table 1:** Factor loadings for perpetrator A and B, item means, and item-total correlations.

		Perpetrator A			Perpetrator B		
Item	Description	Λ	M (SD)	r_it_	Λ	M (SD)	r_it_
	*Module 1*						
**1**	explain to the child why he/ she did something wrong ^b^	.08	2.81 (1.27)	-	-	2.65 (1.33)	-
**2**	put the child in “time out” or send him/ her to his/ her room	.24	1.26 (1.28)	.24	.29	.89 (1.19)	.30
**3**	shake the child	.30	.17 (.59)	.28	.34	.16 (.60)	.33
**5**	give the child something else to do	.11	1.14 (1.25)	.14	.17	.95 (1.23)	.21
**6**	shout, yell or scream at the child	.48	.94 (1.17)	.45	.57	.91 (1.16)	.51
**8**	spank the child	.52	.25 (.74)	.39	.49	.23 (.72)	.41
**10**	say bad words to the child	.59	.31 (.75)	.54	.59	.25 (.69)	.50
**12**	tell the child to be sent away or kicked out of the house	.48	.20 (.67)	.42	.41	.12 (.54)	.37
**14**	threaten the child with spanking or hitting	.57	.23 (.67)	.48	.51	.19 (.60)	.42
**16**	slap the child on the hand, arm or leg	.57	.21 (.61)	.49	.59	.18 (.62)	.48
**17**	take away the child’s favorite toy	.34	.49 (.93)	.32	.35	.35 (.82)	.35
**18**	pinch the child when he/ she did something wrong	.47	.10 (.42)	.38	.42	.08 (.42)	.33
**21**	call the child dumb or lazy	.39	.55 (.95)	.38	.42	.40 (.85)	.40
**22**	slap the child on the face	.70	.22 (.65)	.56	.71	.19 (.62)	.56
**7**	punch or kick the child	.65	.08 (.39)	.50	.55	.09 (.43)	.38
	*Module 2*						
**4**	hit the child on the bottom with something hard	.90	.04 (.31)	.70	.80	.04 (.34)	.64
**11**	beat the child up ^a^	.32	.02 (.24)	.70	.76	.03 (.25)	.67
**15**	hit the child with something hard	.92	.03 (.28)	.74	.56	.03 (.30)	.46
**20**	throw or knock the child down	.32	.04 (.25)	.28	.72	.03 (.26)	.56
	*Module 3*						
**9**	grab the child around the neck and choke him/ her	.94	.01 (.09)	.71	.85	.01 (.11)	.69
**13**	burn the child on purpose ^b^	.98	.00 (.08)	-	-	.00 (.11)	-
**19**	threaten the child with a knife or a gun	.58	.00 (.05)	.54	.80	.00 (.06)	.69

Λ = factor loadings, M = item mean, r_it_ = corrected item-total correlation.

^a^ Item 11 was shifted from module 2 to module 3.

^b^ Items 1 and 13 were omitted.

#### Child maltreatment status in caregiver reports

The Maternal Interview on Child Maltreatment (MICM) [23] contains ten standardized screen questions to assess lifetime presence and dimensions of different subtypes of child neglect and maltreatment in a personal interview with the caregiver. In the present study, physical abuse, emotional maltreatment, sexual abuse and neglect (i.e. failure to provide and lack of supervision) were included in the analyses. Trained researchers rated maltreatment incidents using the manualized Maltreatment Classification System (MCS) [24], a reliable and valid coding system (e.g. [25,26]) that differentiates between maltreatment subtypes by providing mutually exclusive subtype criteria and anchor examples. Coders rated each maltreatment incident that was described during administration of the MICM in terms of subtype, severity (1 = low to 5 = high), and developmental period in which the incident occurred, i.e. infancy (0–1.4 years), toddlerhood (1.5–2 years), preschool age (3–5 years), early school age (6–7 years), late school age (8–12 years), and adolescence (13–18 years). Two dimensions (chronicity, maximum severity) were derived. To yield an age-independent index of maltreatment chronicity, we computed the percentage of periods affected by maltreatment (affected periods relative to the total number of developmental periods of a child). Agreement on the identification of maltreatment subtypes based on 20% double-coding by six independent raters was high (Cohen’s κ between .78 and 1.00), and inter-rater reliability (intraclass correlation, ICC) on the two dimensions of severity (ICC = .93) and chronicity (ICC = .92) was excellent. In addition, the caregiver answered if there had been any lifetime contact to the Youth Welfare Office.

#### Child symptoms

The Child Behavior Checklist (CBCL/4-18) [27] was used to assess caregivers’ report of the frequency of 115 problem behaviors exhibited by their 4 to 18-year-old child in the past six months. Respondents rate each behavior on a three-point scale (0 = “not true”, 1 = “somewhat or sometimes true”, or 2 = “very true or often true”). This measure has established excellent reliability and validity. In the present study, internalizing and externalizing symptoms were analyzed (Cronbach’s α = .89 and .93).

## Results

### Factor analysis

The 22 items of the CTSPC-R (see Table 1) were submitted to a confirmatory factor analysis (CFA) using LISREL 9.1 to examine the factor structure of the instrument. We compared four models in the perpetrator A sample. For the best fitting model, we attempted to cross-validate this model in the perpetrator B sample. The first model is a one-factor model, where all items belong to one factor without distinguishing between modules with different severity. The second model is a correlated three-factor model, with one factor consisting of nonviolent discipline, psychological aggression, and less severe physical maltreatment (15 items, module 1), a moderate severity factor (4 Items, module 2), and a high severity factor (3 Items, module 3). The third model is a second-order factor model assuming that the three factors of model 1 are highly correlated, and the high correlation is explained by a latent second-order factor. The fourth model is a bi-factor model. This model contains one general factor and three group factors, which represents the above mentioned three factors. Each item is specified to load on the general factor and on one nested factor directly. Substantively, the general factor represents the conceptually broad construct an instrument was designed to measure, and the group factors represent more conceptually narrow subdomain constructs.

Several fit-indices were examined to evaluate the overall fit of the model. Beyond the chi-square goodness-of-fit statistic, the comparative fit index (CFI), and the Tucker-Lewis index (TLI) were used. CFI and TLI are incremental indices reflecting the improvement in fit gained by a given factor model relative to the most restrictive (null or independence) model. Hu and Bentler [28] advised that values close to .95 are indicative of a good fit. Furthermore, we utilized the Standardized Root Mean Square Residual (SRMR), which is an absolute measure of fit and is defined as the standardized difference between the observed correlation and the predicted correlation. A value less than .08 is generally considered as a good fit [28]. The root mean square error of approximation (RMSEA) is a population discrepancy function that compensates for the effects of model complexity. According to Browne and Cudek [29], a RMSEA value of .05 or less indicates a close fit of the model in relation to the degrees of freedom, whereas a value of .08 or less indicates a reasonable error of approximation. The p of Close Fit (PCLOSE) is a one-sided test of the null hypothesis that the RMSEA equals .05. If p is greater than .05, then it is concluded that the fit of the model is "close." Finally, we report two information criteria, AIC and BIC to select the best fitting model.

Results for the four models are reported in Table 2. The fit of the one-factor model is poor, as indicated by inadequate high values for RMSEA and SRMR and inadequate low values for CFI and TLI. The fit for the three-factor model is also poor according to the above-mentioned criteria. The second-order factor model is mathematically equivalent to the correlated three-factor model. Since the latent correlations between the three factors are moderate, we prefer the second-order factor model over the correlated three factor model, because it is sensible to assume that the three factors constitute a higher order factor ranging from nonviolent discipline to highly severe physical abuse. The bi-factor model shows the lowest AIC and BIC and better values for SRMR, CFI, and TLI; whereas the RMSEA was worse compared to the preceding model. However, inspection of the factor loadings reveals five Heywood-cases for the global factor, indicating that estimated variances of these items are negative, and this model shows a misfit. Therefore, we prefer the second-order model as best approximation of the data.

**Table 2:** Fit indices for different models based on maximum likelihood estimation with robust (Huber-White) standard errors and Yuan-Bentler test statistic.

Model	*χ*^2^	df	p	RMSEA	95%CI RMSEA	p-close	SRMR	CFI	TLI	AIC	BIC
**Single Factor Model**											
Perpetrator A	1380.51	209	< .001	.142	.135 - .149	< .001	.095	.438	.379	21456.45	21772.30
**3-Factor Model**											
Perpetrator A	1005.87	206	< .001	.066	063 - .069	< .001	.092	.759	.730	19070.61	19400.81
**2**^**nd**^**-Order Factor Model**											
Perpetrator A	1005.87	206	< .001	.066	063 - .069	< .001	.092	.759	.730	19070.61	19400.81
Perpetrator B^a^	385.01	167	< .001	.039	.036 - .042	1.00	.063	.846	.825	19278.04	19577.95
**Bi-factor Model**											
Perpetrator A	1067.82	185	< .001	.072	.068 - .076	< .001	.066	.887	.859	18347.91	18780.53

Perpetrator A: n = 885, perpetrator B: n = 863.

^a^ Model with item 1 and 13 omitted, and item 11 on module 3 instead of module 2.

### Model modification

**Fig 1:**
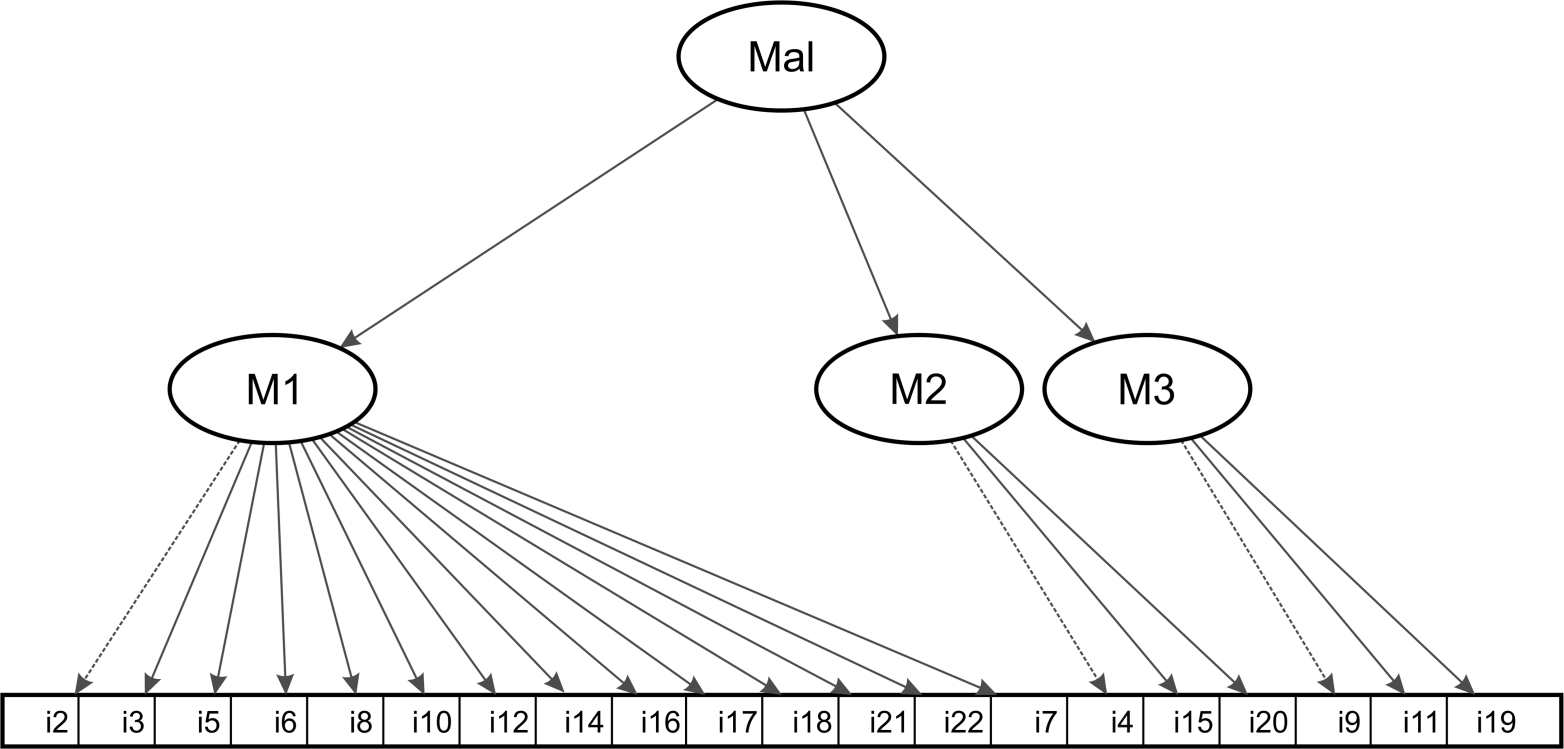
Final model with three correlated first-order factors and a second-order factor. Mal = general factor on child psychological and physical abuse; M1 = module 1, M2 = module 2, M3 = module 3; i = CTSPC-R items.

Before cross-validating the second-order model, we computed modification indices (mi) to understand the just moderate model fit of the second-order model. The largest mi (307.01) suggests that item 11 (“beat the child up”) should be moved from the moderate severity factor (module 2) to the high severity factor (module 3). As regard to content, the shift from moderate to severe physical abuse is justified. Therefore, for the cross-validation of the second-order model we shift item 11 to the high severity factor. Furthermore, item 1 (“explain to the child why he/ she did something wrong”) shows a very low factor loading (.09) on the minor severity factor (module 1), thus this item was omitted from further analyses. Finally, as item 9 (“grab the child around the neck and choke him/ her”) and 13 (“burn the child on purpose) from the severe physical abuse factor are highly correlated not only for perpetrator A (*r* = .93), but also for perpetrator B (*r* = .95), we decided to omit item 13 on base of the Mean Absolute Deviation (MAD), which is slightly larger for item 9 (.011) than for item 13 (.009) for both perpetrators. The final model which is illustrated in Fig 1 includes 20 items with 14 items loading on module 1, and module 2 and 3 comprising 3 items each. Factor loadings for perpetrator A and B, item-total correlations, and item means of the final factor solution are presented in Table 1.

The fit of the modified model is very good in terms of low SRMR and RMSEA, and moderate for CFI and TLI (see Table 2). Factor loadings for the first-order factors ranged from .11 to .94 for perpetrator A, and from .17 to .85 for perpetrator B (see Table 1). Loadings on the second-order factor in perpetrator B were .88, .68, and .41 for the first, second, and third first-order factor, respectively.

### Internal consistency

Coefficients of internal consistencies of the first-order factors and the second-order factor are displayed in Table 3. The Cronbach’s α coefficient for the first-order factors ranged from .66 to .75, whereas McDonalds’ Ω ranged from .74 to .78. For the second-order factor, α was .77, and Ω was .78.

**Table 3:** Reliability measures for the three modules and the second-order factor in perpetrator B.

	M1	M2	M3	Second-order factor
**Cronbach’s Alpha**	.75	.72	.66	.77
**McDonald’s Omega**	.75	.74	.78	.78

M1 = Module 1, M2 = Module 2, M3 = Module 3.

### Construct and concurrent validity

As we found evidence for a higher order factor ranging from nonviolent discipline to high severity physical abuse, we created a CTSPC-R severity scale. Therefore, absence and presence of maltreatment in each CTSPC-R module was treated as one point of an ordinal Likert scale (0 = absence of maltreatment, 1 = presence of maltreatment in module 1, 2 = presence of maltreatment in module 2, 3 = presence of maltreatment in module 3). The construct validity of the CTSPC-R was assessed by calculating correlations between CTSPC-R severity in the caregiver dyad (perpetrator A and/or B) and lifetime CPS contact as well as presence of maltreatment subtypes in caregiver-report (i.e. physical abuse, emotional maltreatment, failure to provide, lack of supervision, and sexual abuse). Cramer’s *V* was 0.147 (*p* < .001) for lifetime CPS contact, 0.188 (*p* < .001) for caregiver-reported physical abuse, and 0.112 (*p* < .05) for caregiver-reported emotional maltreatment, indicating significant but small associations. The correlations were not significant for caregiver-reported failure to provide (Cramer’s *V* = 0.062, *p* = 0.335), lack of supervision (Cramer’s *V* = 0.078, *p* = 0.154), and sexual abuse (Cramer’s *V* = 0.054, *p* = 0.465).

Moreover, Spearman’s correlation coefficient revealed small, significant associations of CTSPC-R severity in the caregiver dyad with caregiver-reported severity and chronicity of physical abuse (*r*_*s*_ = .139 and *r*_*s*_ = .121, *p*s < .001, respectively) and emotional maltreatment (*r*_*s*_ = .092, *p* < .01, and *r*_*s*_ = .086, *p* < .05, respectively).

Moreover, to test for concurrent validity, we compared mean scores of child symptoms between subgroups based on maltreatment reports on the CTSPC-R (No Mal = absence of maltreatment, M1 = presence of maltreatment in module 1, M2 = presence of maltreatment in module 2, M3 = presence of maltreatment in module 3) by computing a MANOVA. We included risk-setting (i.e. community sample/s1 vs. non-community sample/s2+s3) as a second factor and tested for interactions between CTSPC-R groups and risk-setting. Multivariate results showed significant main effects for CTSPC-R groups (Wilks-Lambda = 0.95, *p* < 0.001, η_p_^2^ = 0.02) and risk-setting (Wilks-Lambda = 0.97, *p* < 0.001, η_p_^2^ = 0.03) on child symptoms, but no significant interaction effect (Wilks-Lambda = 1.00, *p* = 0.77, η_p_^2^ = 0.00). According to the univariate results, the significant main effects were due to internalizing and externalizing symptoms. Post hoc tests (Games-Howell) for CTSPC-R groups (see Table 4) indicated that for internalizing symptoms, the M3 group showed higher scores than the M2 group, and the M2 and M3 groups both showed higher scores than the No Mal group, whereas there was no significant difference between M1 and No Mal or between M2 and M3. For externalizing symptoms, the M2 and M3 groups both differed significantly from M1 and No Mal, whereas there was no significant difference between M1 and No Mal. Post hoc tests for risk-setting revealed significantly higher externalizing (*F*(1, 803) = 23.64, *p* < 0.001, η_p_^2^ = 0.03) and internalizing symptoms (*F*(1, 803) = 8.68, *p* < 0.01, η_p_^2^ = 0.01) in the non-community sample compared to the community sample.

**Table 4:** Means and standard deviations for child symptoms by CTSPC-R prevalence in the caregiver dyad (perpetrator A+B).

	No Mal (n = 62)	M1 (n = 701)	M2 (n = 36)	M3 (n = 12)
**Int symp**	7.53 (5.66)	8.66 (7.17)	12.42 (8.63)	16.41 (9.13)
**Ext symp**	7.77 (7.00)	10.30 (9.20)	17.56 (10.08)	20.92 (9.53)

No Mal = No Maltreatment, M1 = Module 1 maltreatment, M2 = Module 2 maltreatment, M3 = Module 3 maltreatment.

F-values (*F*) and effect sizes (η_p_^2^) of ANOVAS: internalizing symptoms (int symp) *F*(3, 803) = 5.67, *p* < 0.01, η_p_^2^ = 0.02; externalizing symptoms (ext symp) *F*(3, 803) = 11.91, *p* < 0.001, η_p_^2^ = 0.04.

### Age and gender analysis

Next, we analyzed influences of child age and gender on mean differences in child symptoms between CTSPC-R subgroups. Multivariate results showed significant main effects for child age group (Wilks-Lambda = 0.99, *p* < 0.01, η_p_^2^ = 0.01), gender (Wilks-Lambda = 0.99, *p* < 0.05, η_p_^2^ = 0.01), and CTSPC-R groups (Wilks-Lambda = 0.96, *p* < 0.001, η_p_^2^ = 0.02), but no significant interaction effects. Post hoc differences for CTSPC-R groups remained significant. For internalizing symptoms, the M3 group showed higher scores than the M2 group, and the M2 and M3 groups both showed higher scores than the No Mal group. For externalizing symptoms, the M2 and M3 groups both showed higher scores than the M1 and the No Mal groups. The univariate results revealed significant effects of age (*F*(1, 799) = 9.18, *p* < 0.01, η_p_^2^ = 0.01), and gender (*F*(1, 799) = 4.30, *p* < 0.05, η_p_^2^ = 0.01) on internalizing symptoms, with older and female children providing higher symptoms. No effects of age and gender on externalizing symptoms could be found.

## Discussion

Our study provides a psychometric evaluation of a child-report measure of psychological and physical abuse appropriate for a broad age-range from 4 to 16 years in a heterogeneous sample of community and at-risk families. Our study therefore broadens our ability to assess children’s appraisal of physical abuse within the family context. For assessing current incidences of psychological and physical assault in a broad age range from early childhood (starting from age 4) to adolescence, we a) developed child-appropriate picture drawings to scaffold a better understanding of item content for young children, and b) implemented a sequential step-by-step, hierarchical presentation of items, arranged in three ascending levels of maltreatment severity. A confirmatory factor analysis supported the three-factor solution of the assumed three modules and revealed a latent second-order factor explaining the correlation between the factors. The second-order latent factor represents the global concept of psychological and physical abuse, and the co-occurrence of maltreatment subtypes, which is common in the literature. Yet, the inter-correlation between the three factors was moderate, indicating that these scales represent sufficiently distinct entities. Children, on the one hand, may have difficulties distinguishing between different subtypes of maltreatment, but, on the other hand, seem to be able to rate the severity of incidences. Further psychometric properties of the factors revealed good internal consistencies (Cronbach’s α between .66 and .75; McDonalds’ Ω between .75 and .78).

In support of convergent validity, CTSPC-R severity was linked to caregiver-report on maltreatment at the global (i.e. CPS-contact) and the subtype-specific level (i.e., emotional maltreatment and physical abuse). Discriminant validity was bolstered by non-significant associations with distinct, caregiver-reported maltreatment subtypes, more specifically neglect (i.e. failure to provide, lack of supervision), and sexual abuse. As a crucial criterion for concurrent validity, subgroups with moderate and severe physical abuse provided more caregiver-reported externalizing and internalizing symptoms, and scores were not moderated by sample type (i.e. community vs. at-risk), child age or gender. These results are in line with previous studies linking self-reports of psychological and physical abuse to psychiatric symptoms in older children and adolescents [11–14], but extend previous studies by validating younger children’s reports.

As a limitation of our study, the fit of the first second-order model was only moderate, so model modification was necessary, including omission of two items and moving one item to another module. The modified, final second-order model showed very good (SRMR) to moderate (RMSEA) fit indices.

In addition, prevalence rates of moderate (module 2: 4.3% and 3.0% for perpetrator A and B) and severe physical abuse (module 3: 1.5% and 1.6% for perpetrator A and B) were relatively low, and thus, the distribution of the scales was skewed, a psychometrical challenge that has been reported in previous studies on physical abuse [10,15,16]. One possible reason could be that children and adolescents may be reluctant to report on these very severe, traumatic maltreatment experiences. Another explanation may be that these incidences occur very rarely. Supporting this explanation, Straus et al. [10] reported lifetime prevalence rates of one per thousand for burning or threatening the child, and seven per thousand for choking the child. Notably, however, our study included a subsample of CPS-referred children who are at heightened risk of such rare occurrences.

Another issue is the higher risk of social desirability in self-reports compared to formal documentations. Although the CTS and its adaptations are able to reveal more cases of child abuse than CPS-reports [9], these prevalence rates are typically still seen as lower bound estimates of physical abuse in the population, as victims, especially children, may have qualms regarding disclosure of abuse by their caregivers. Moreover, cultural diversity of illustrations could not be achieved, and possible effects of race or ethnicity were not assessed in the present study.

Further studies will be required to test the temporal stability of the scales or the overlap with other child-report instruments on physical maltreatment. Cross-cultural comparisons will also be necessary, because normative issues and legality of corporal punishment of children varies between countries, which may result in different disclosure rates of physical abuse in families.

In sum, our data on the factor-structure, internal consistency, and validity corroborate the CTSPC-R as a promising child-report measure to capture their experiences of psychological and physical abuse across a broad age range, and in high-risk and low-risk populations. Our newly implemented picture battery as well as our modularized sequential presentation aims to do justice to some of the difficulties of assessment in this area, especially for young children. Consequently, the CTSPC-R can be beneficial in maltreatment research on low-risk and high-risk samples and is also applicable in clinical settings as a developmentally sensitive screening tool assessing severity of psychological and physical abuse from the children’s perspective. Related to this, the CTSPC-R can also be used for the evaluation of prevention and intervention following child maltreatment. Hence, we believe this instrument will fill an important gap in the literature, taking us a step closer to meeting the need for the “child’s right to be heard”.

## Supporting information

S1 File **Figures A-U. Pictures of CTSPC items.** This article should be cited when using the images. A more detailed guide can be requested from the authors.
(DOCX)
