# Supplementary material for: Assessing psychological and physical abuse from children’s perspective: Factor structure and psychometric properties of the picture-based, modularized child-report version of the Parent-Child Conflict Tactics Scale – Revised (CTSPC-R)
Source: PLoS One. 2018 Oct 8;13(10):e0205401. doi: 10.1371/journal.pone.0205401 (PMC6175525; doi:10.1371/journal.pone.0205401)
Supplement: S1 File — Figures A-U. Pictures of CTSPC items. This article should be cited when using the images. A more detailed guide can be requested from the authors. (DOCX) [file pone.0205401.s001.docx]

S1 File


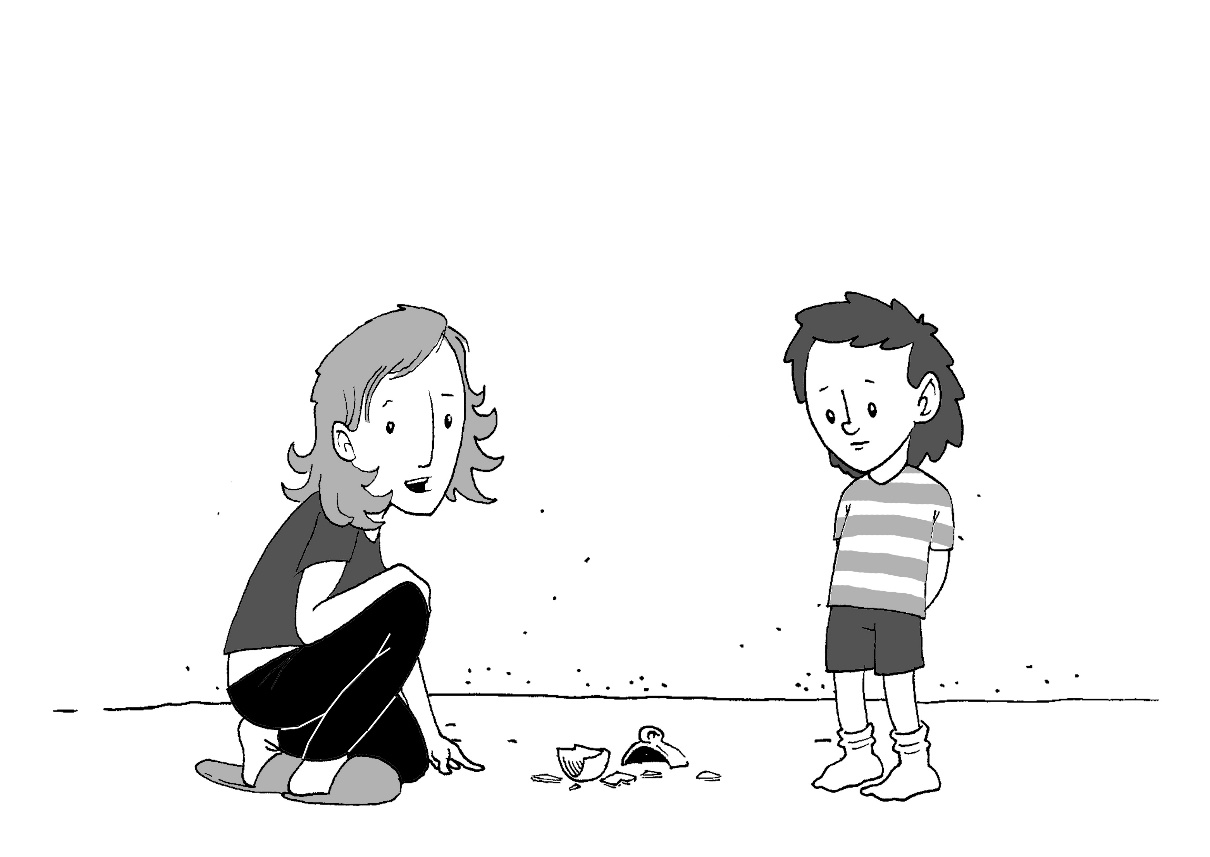


**Figure A1. Item 1(target-parent: mother): “explain to the child why he/ she did something wrong”.**


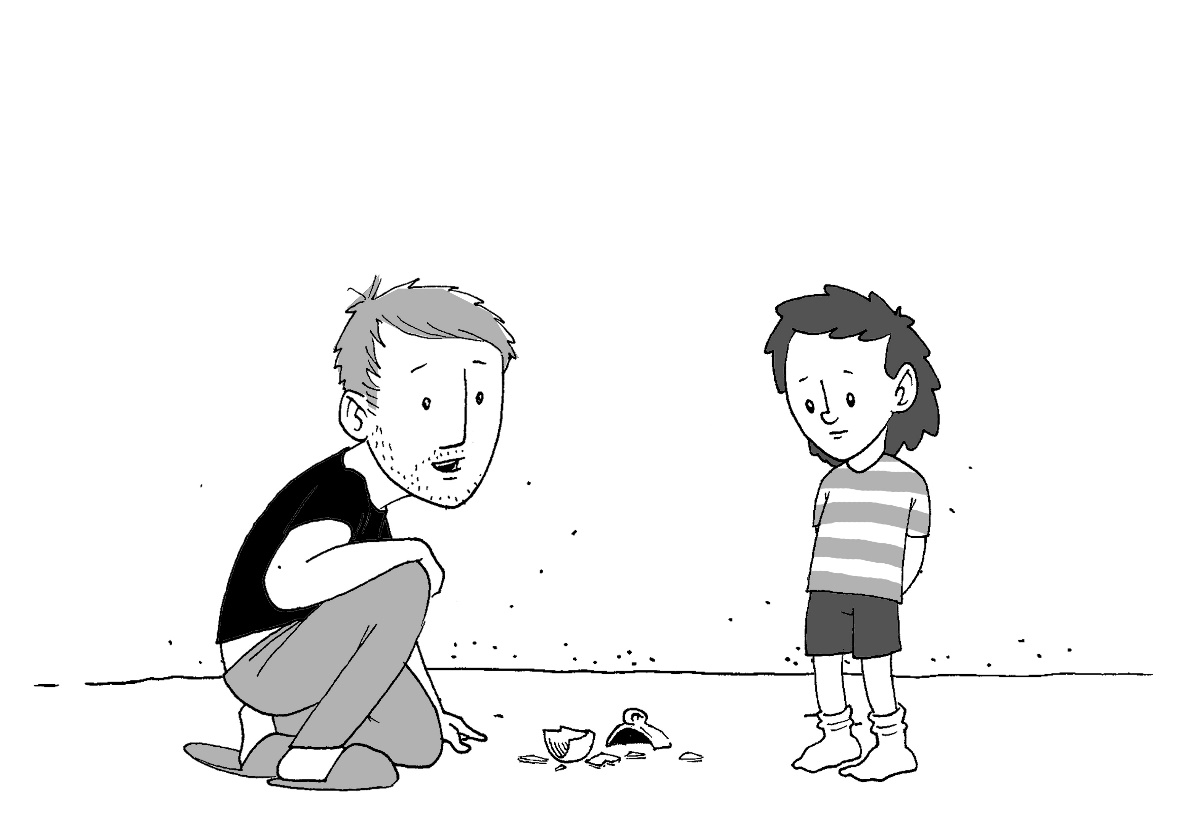


**Figure A2. Item 1(target-parent: father): “explain to the child why he/ she did something wrong”.**


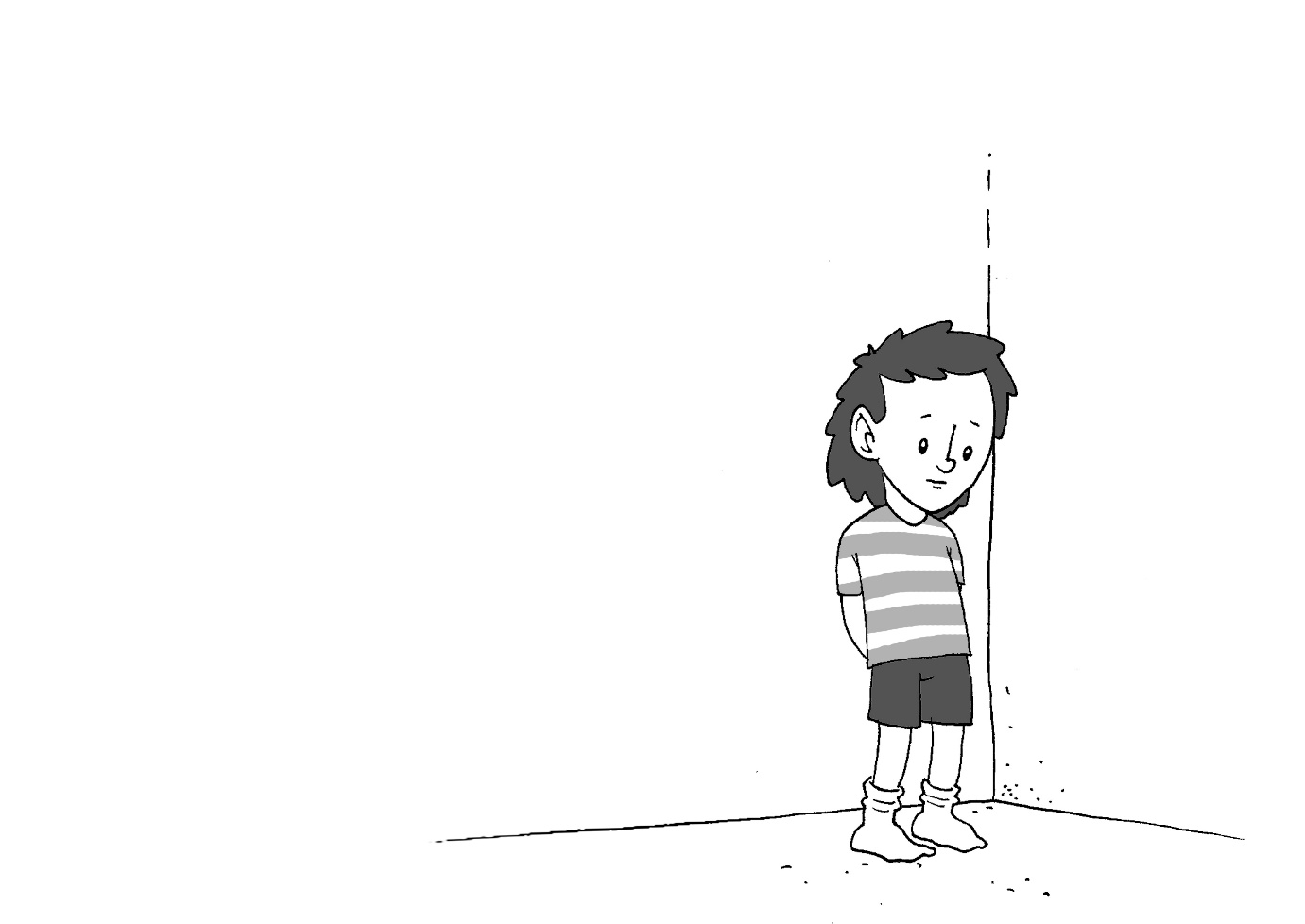


**Figure B. Item 2: “put the child in “time out” or send him/ her to his/ her room”.**


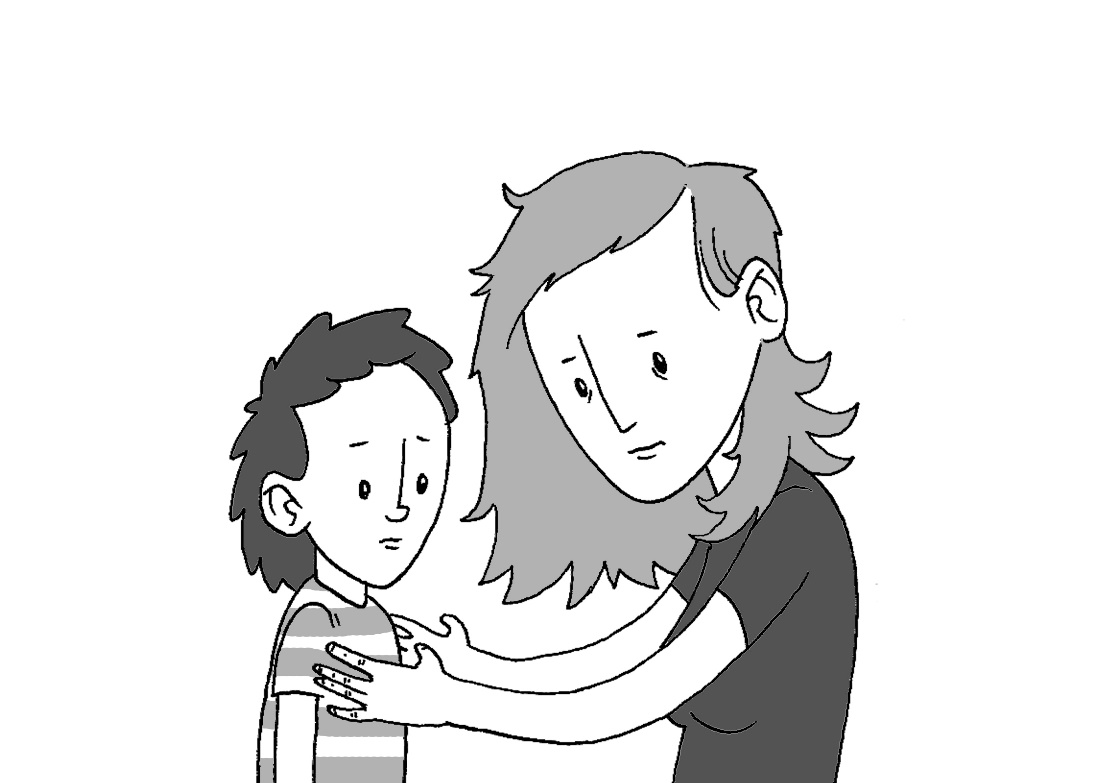


**Figure C1. Item 3** **(target-parent: mother): “shake the child”.**


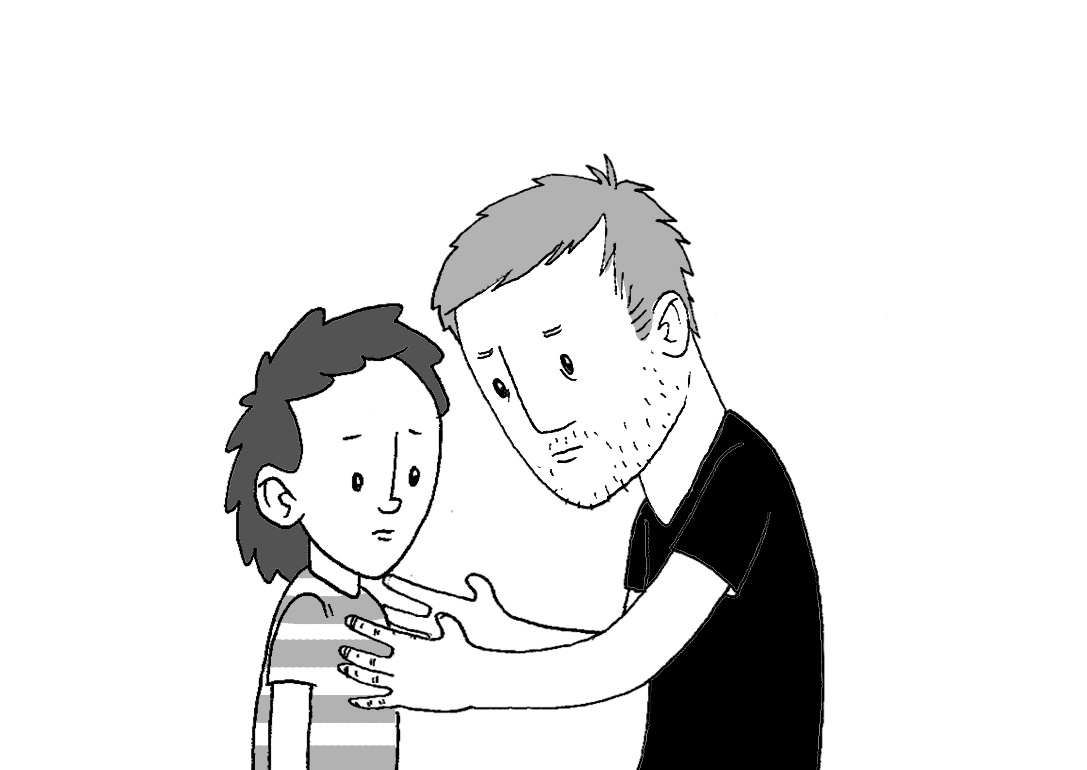


**Figure C2. Item 3 (target-parent: father): “shake the child”.**


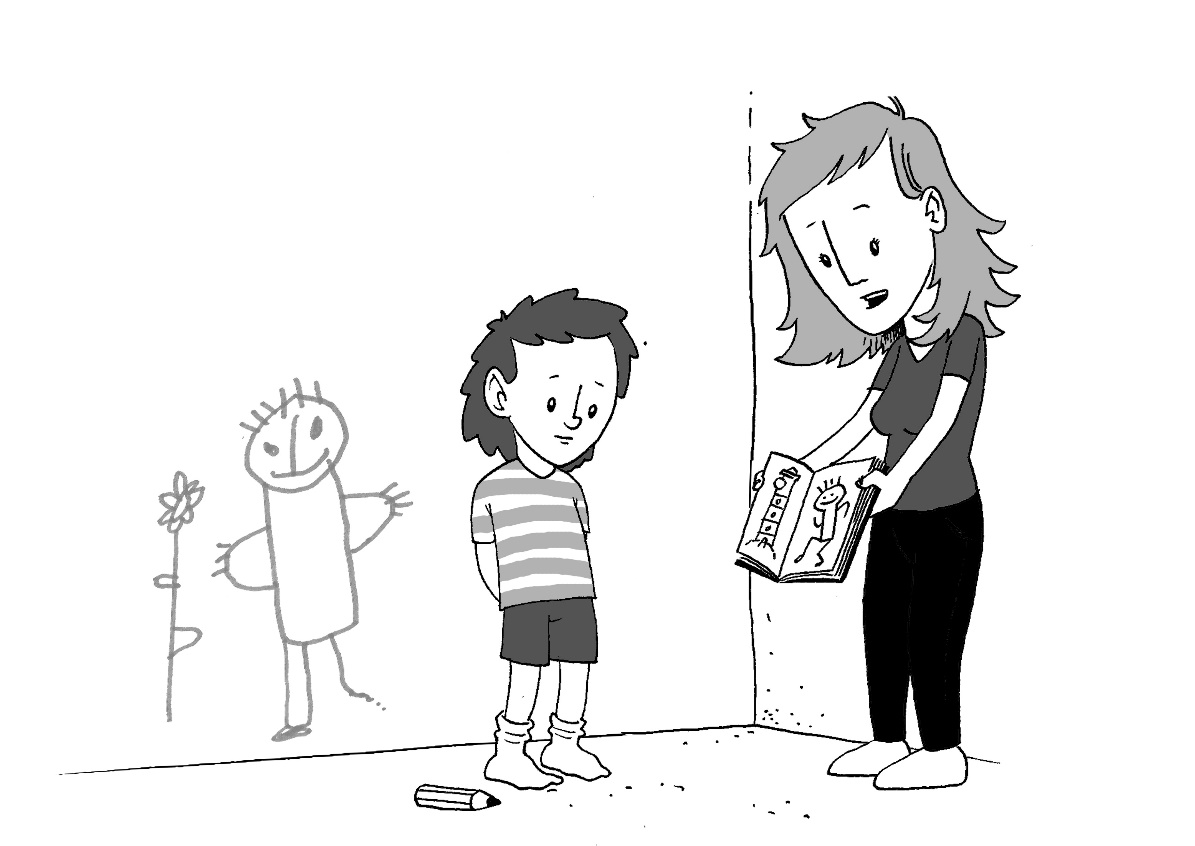


**Figure D1. Item 5 (target-parent: mother): “give the child something else to do”.**


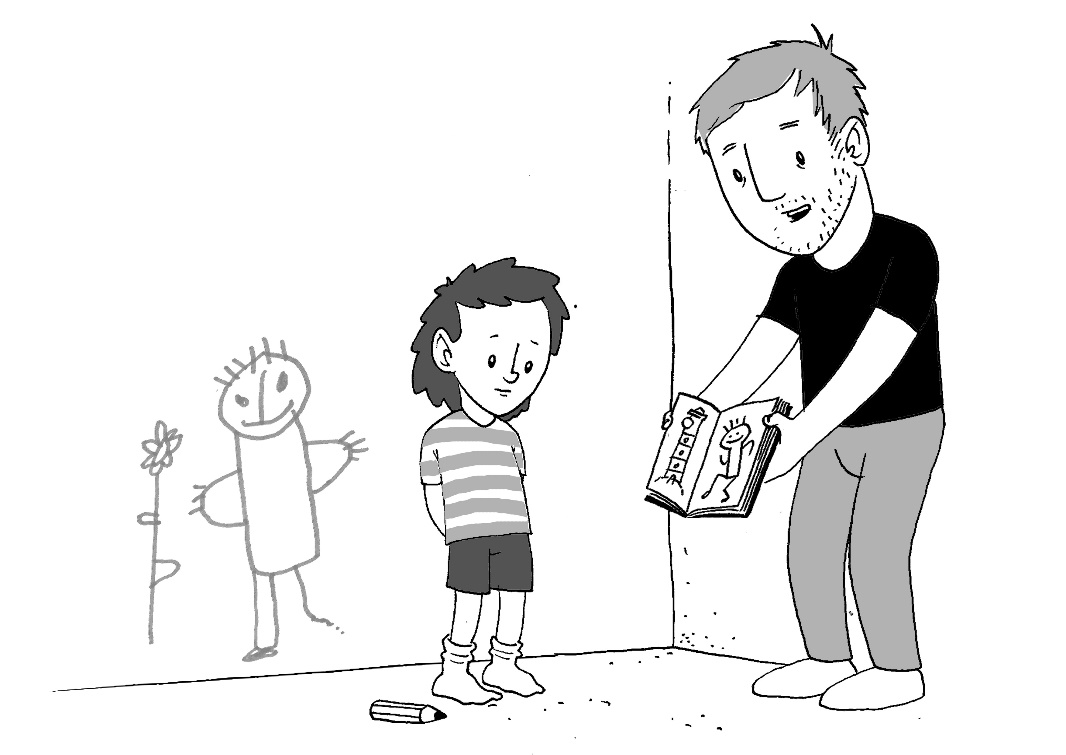


**Figure D2. Item 5 (target-parent: father): “give the child something else to do”.**


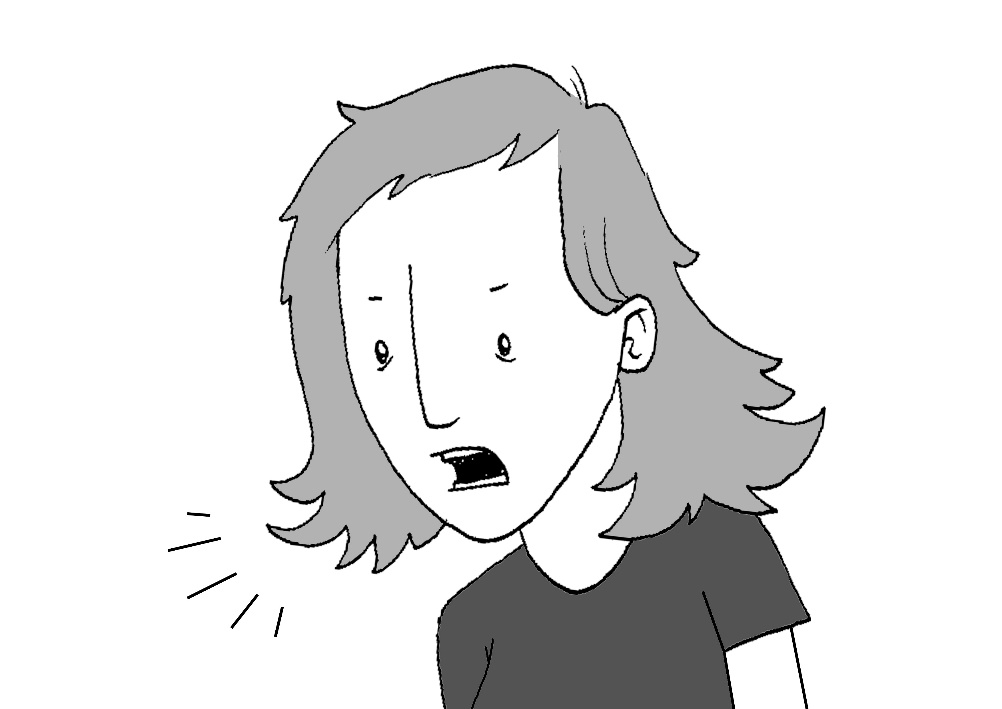


**Figure E1. Item 6 (target-parent: mother): “shout, yell or scream at the child”.**


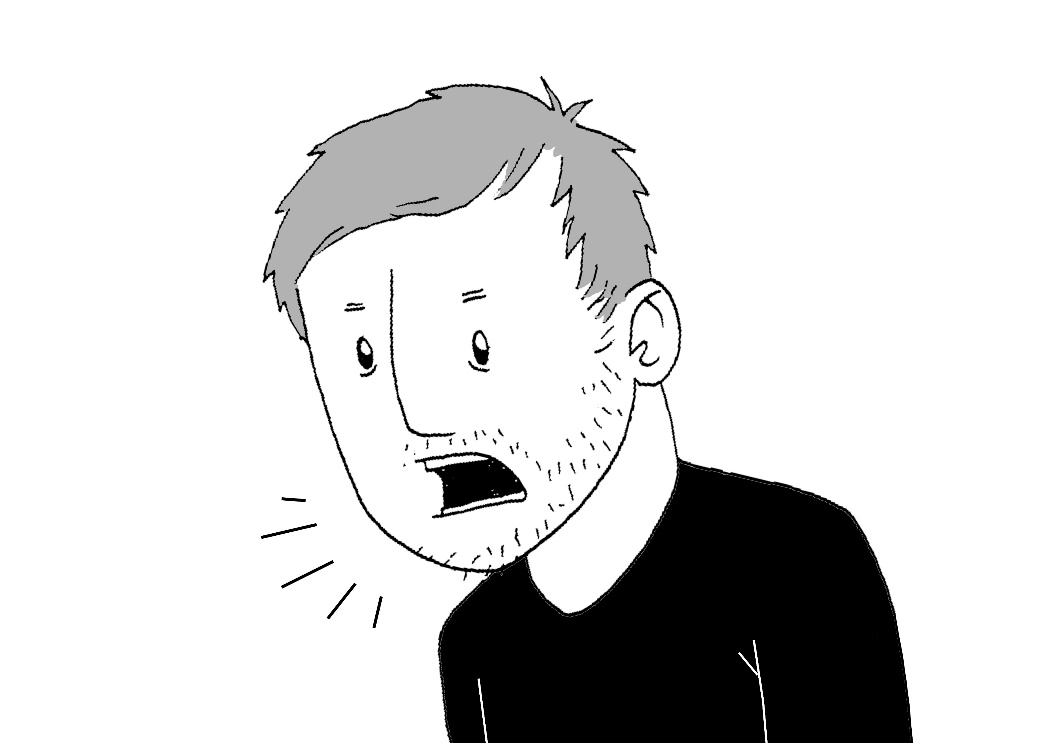


**Figure E2. Item 6 (target-parent: father): “shout, yell or scream at the child”.**


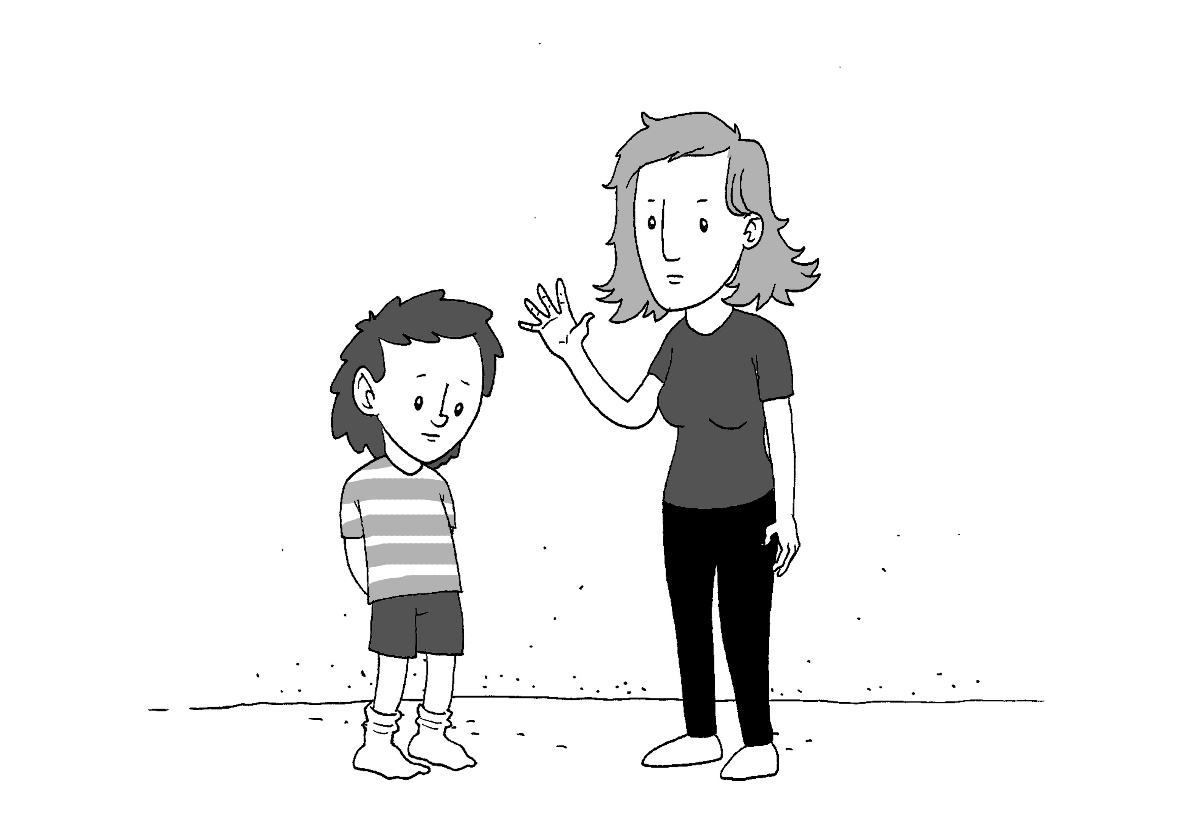


**Figure F1. Items 8 (target-parent: mother): “spank the child”.**


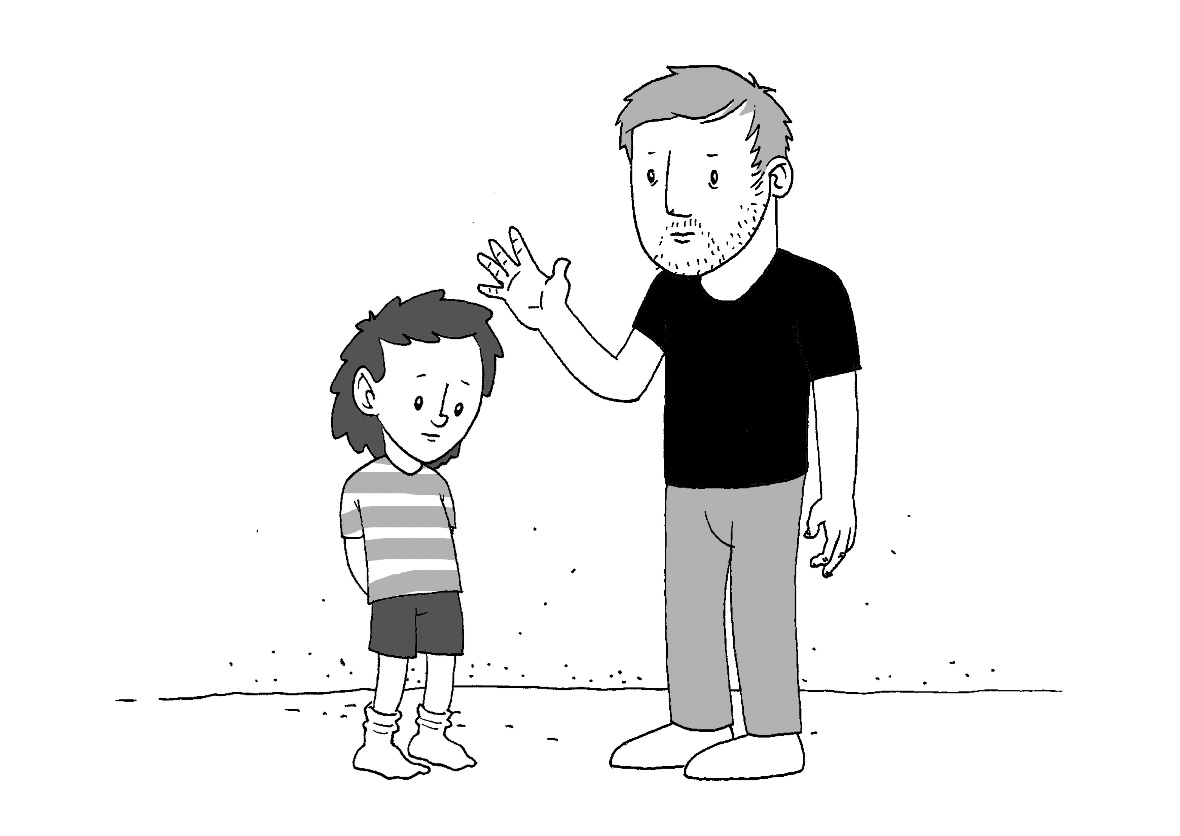


**Figure F2. Items 8 (target-parent: father): “spank the child”.**


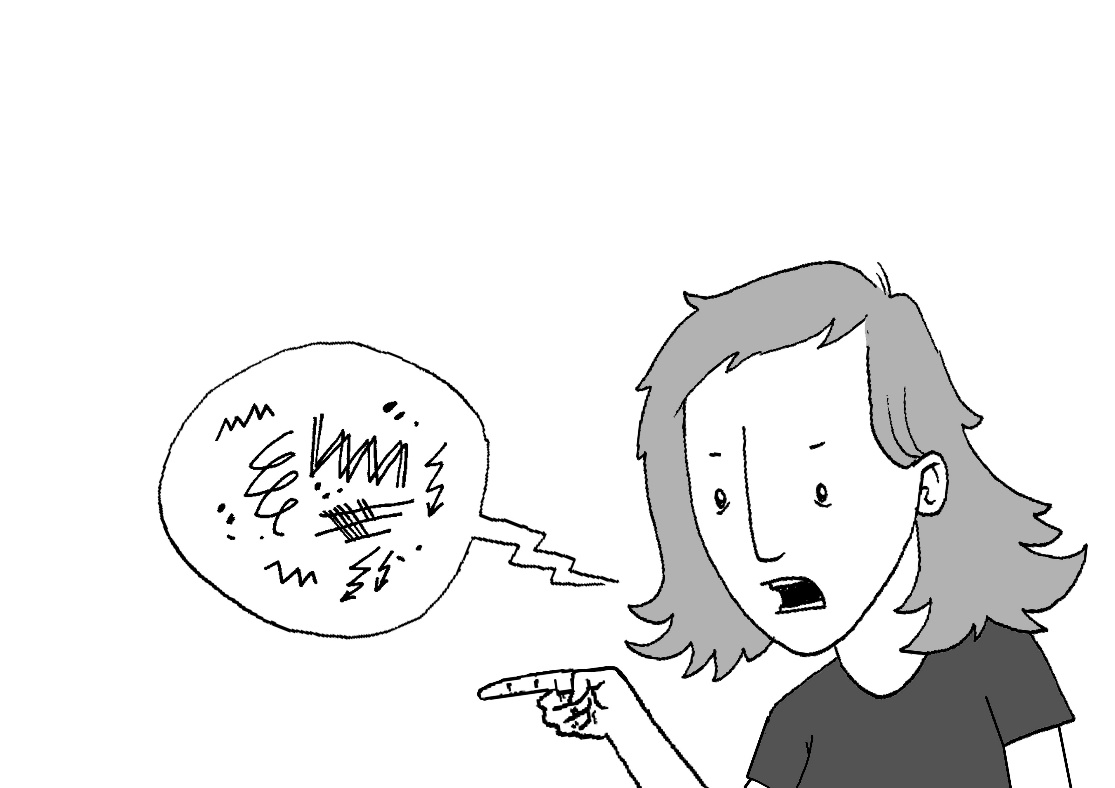


**Figure G1. Item 10 (target-parent: mother): “say bad words to the child”.**


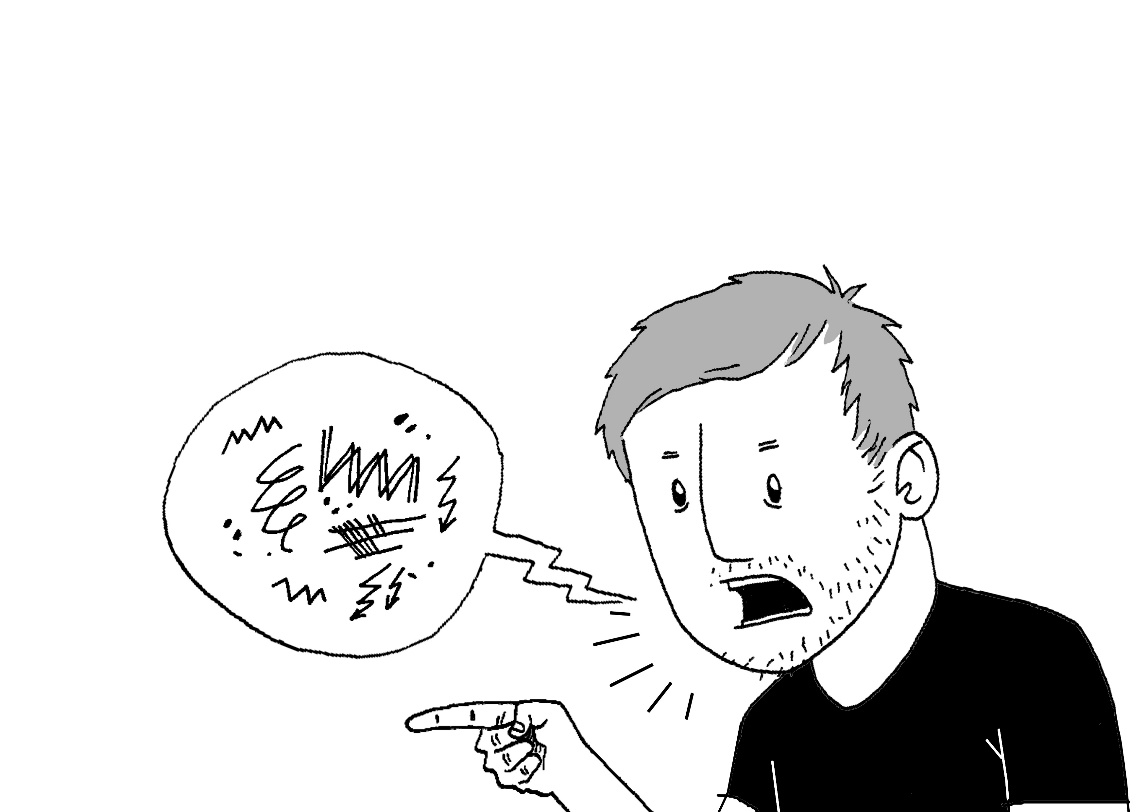


**Figure G2. Item 10 (target-parent: father): “say bad words to the child”.**


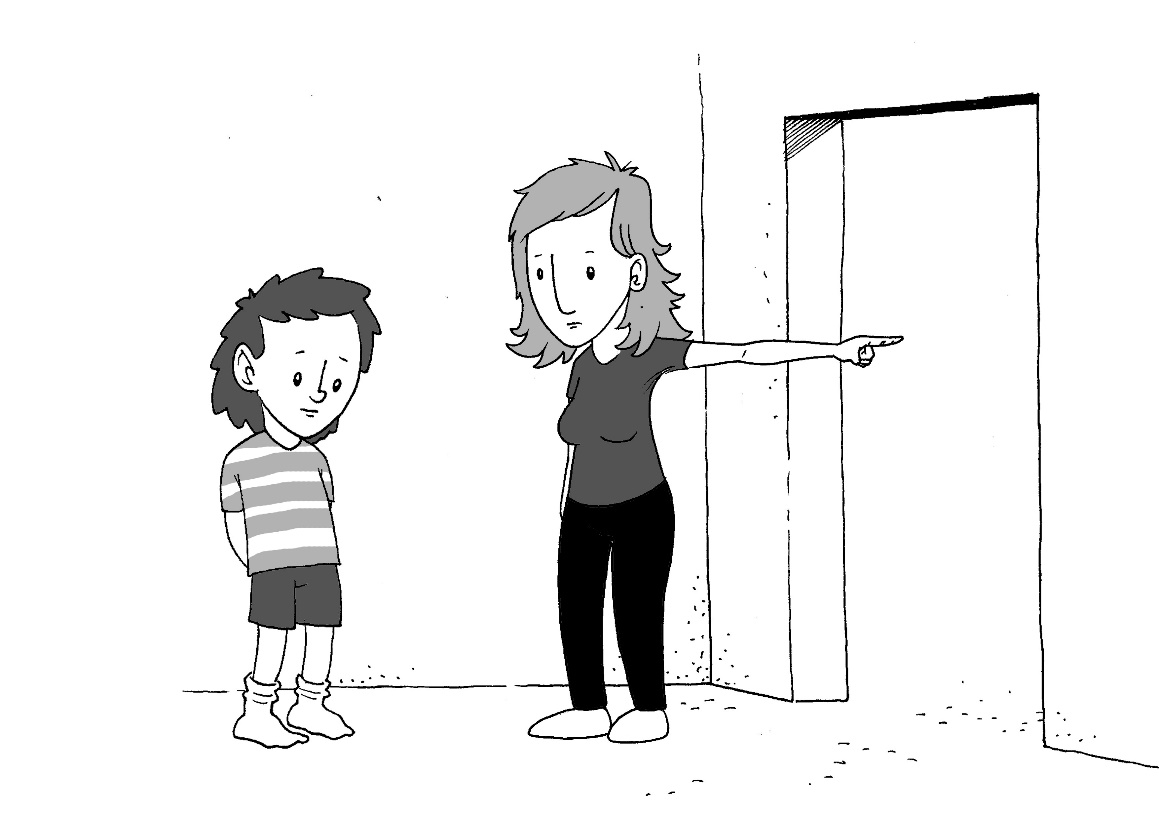


**Figure H1. Item 12 (target-parent: mother): “tell the child to be sent away or kicked out of the house”.**


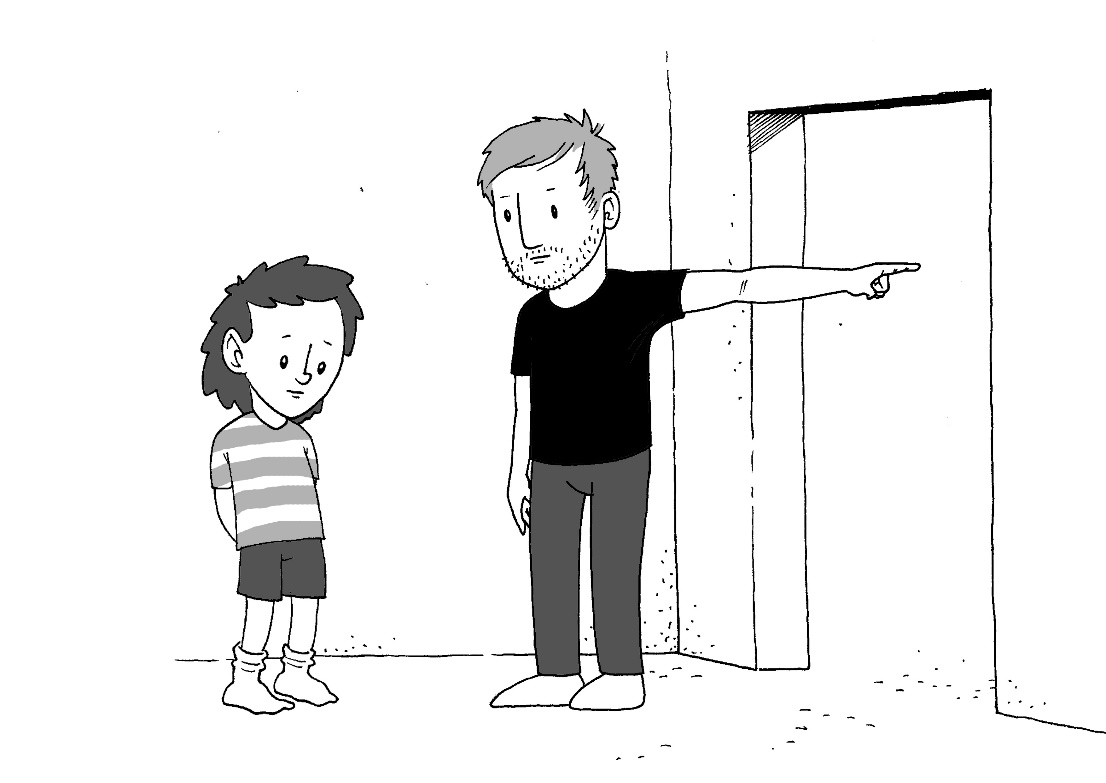


**Figure H2. Item 12 (target-parent: father): “tell the child to be sent away or kicked out of the house”.**


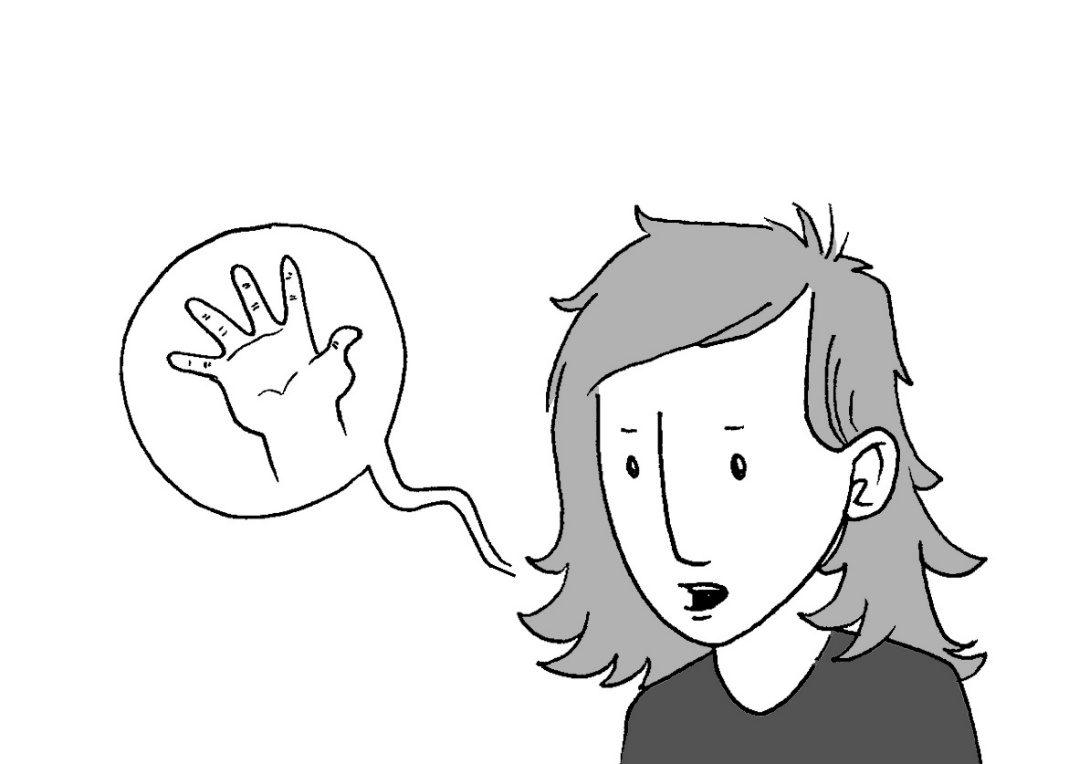


**Figure I1. Item 14 (target-parent: mother): “****threaten the child with spanking or hitting”.**


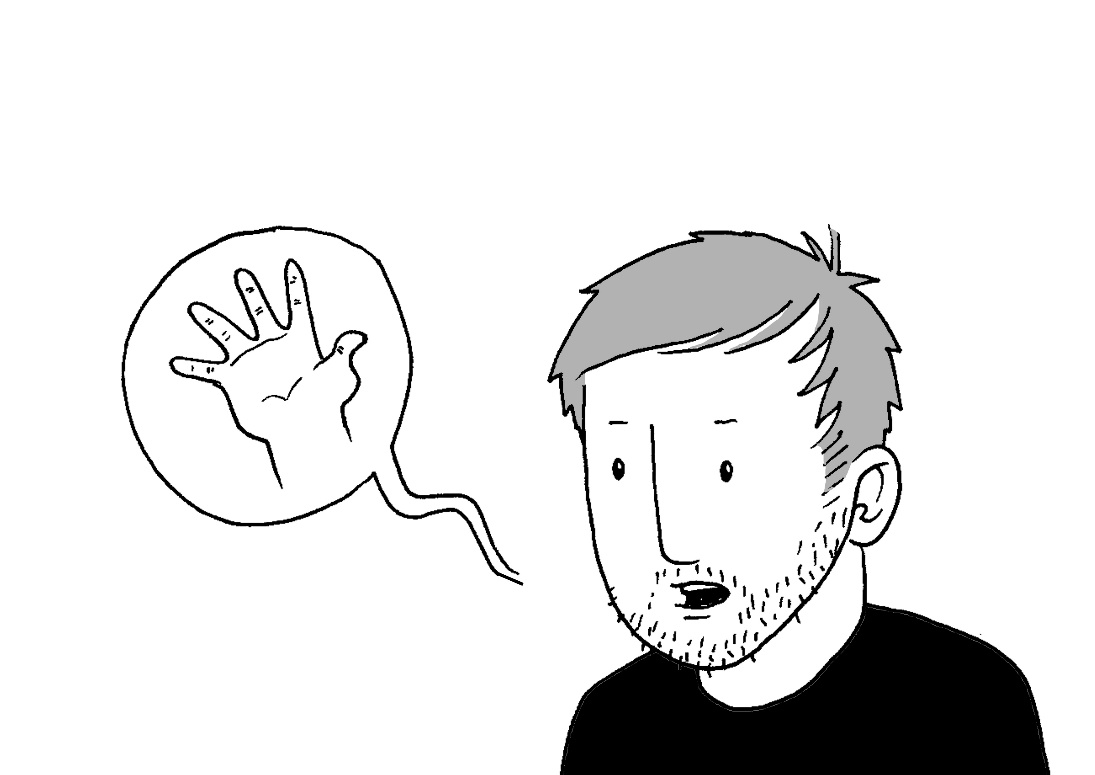


**Figure I2. Item 14 (target-parent: father): “threaten the child with spanking or hitting”.**


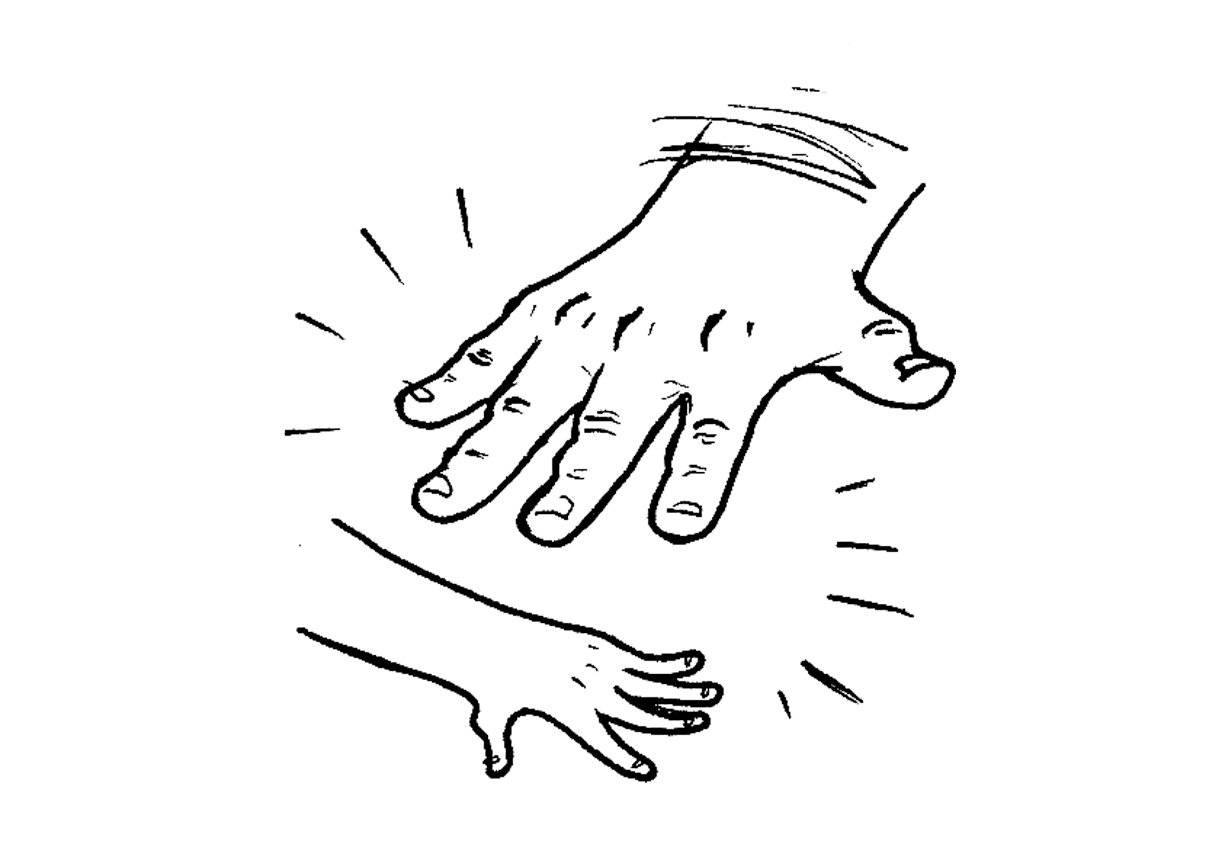


**Figure J. Item 16: “slap the child on the hand, arm or leg”.**


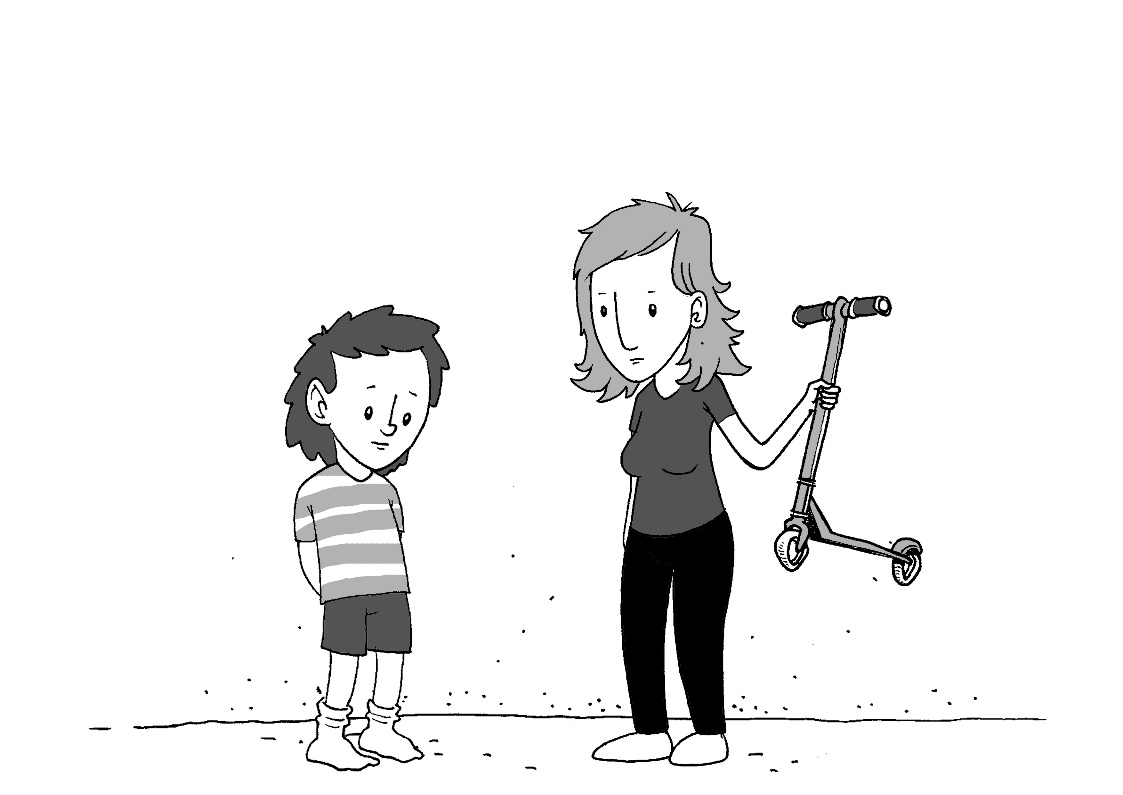


**Figure K1. Item 17 (target-parent: mother): “take away the child’s favorite toy”.**


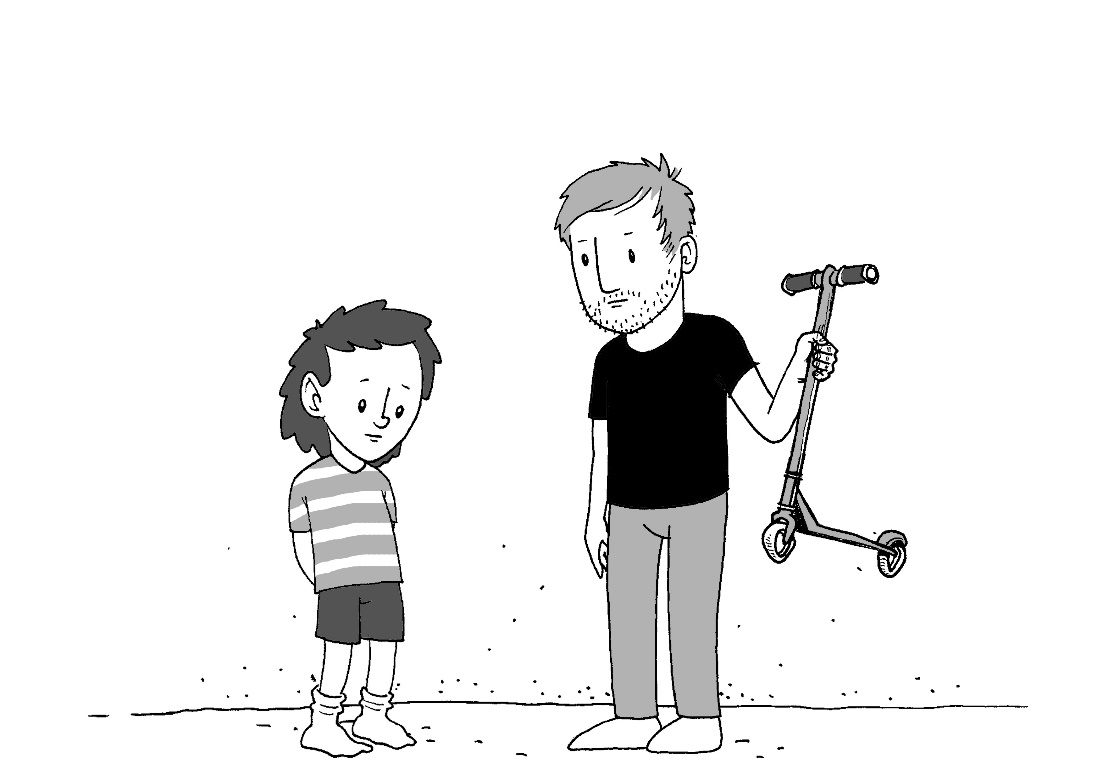


**Figure K2. Item 17 (target-parent: father): “take away the child’s favorite toy”.**


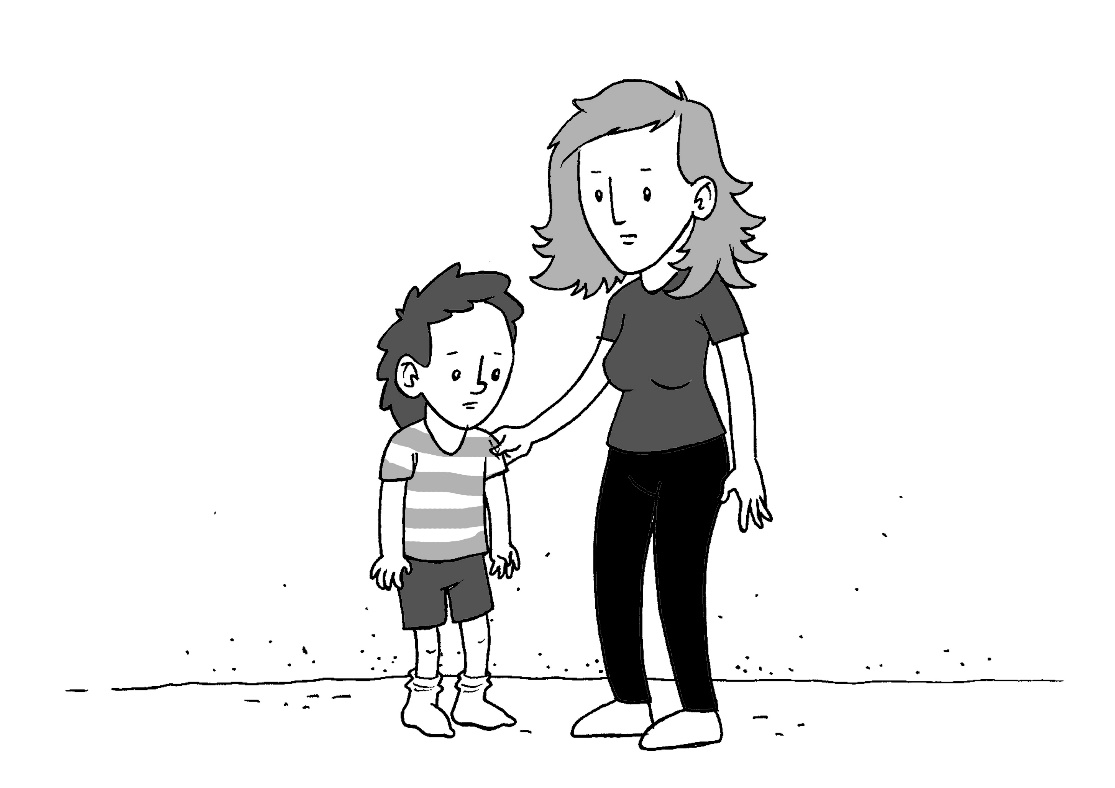


**Figure L1. Item 18 (target-parent: mother): “pinch the child when he/ she did something wrong”.**


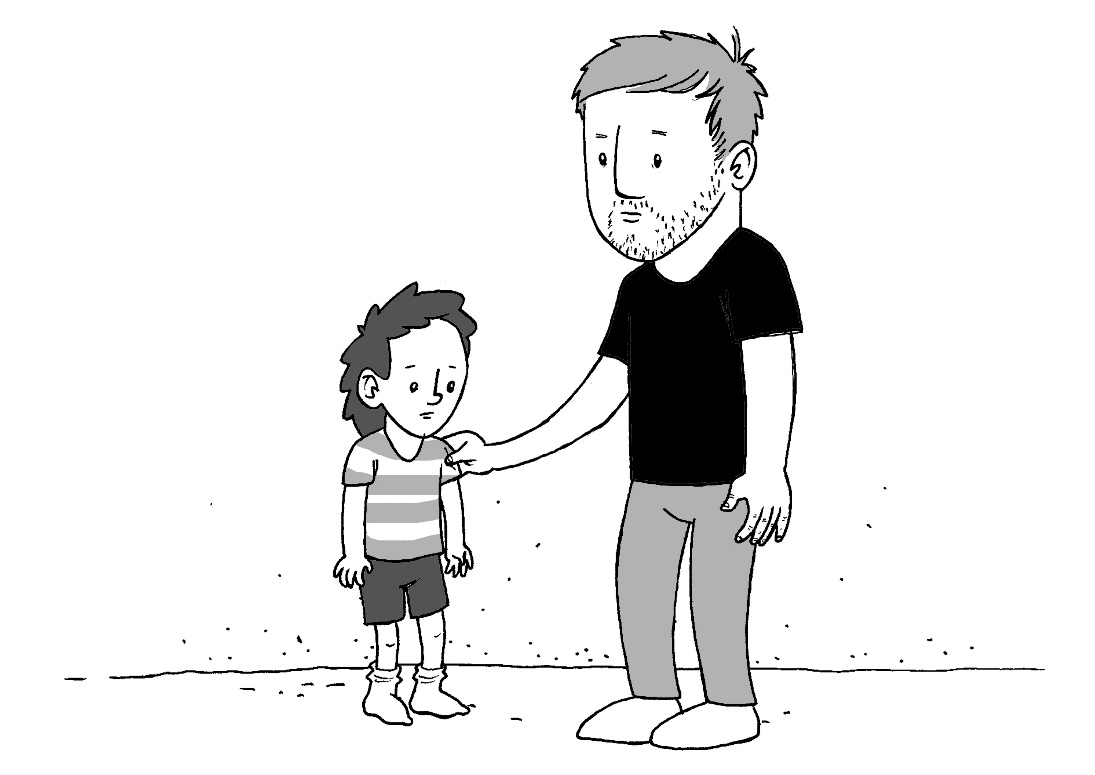


**Figure L2. Item 18 (target-parent: father): “pinch the child when he/ she did something wrong”.**


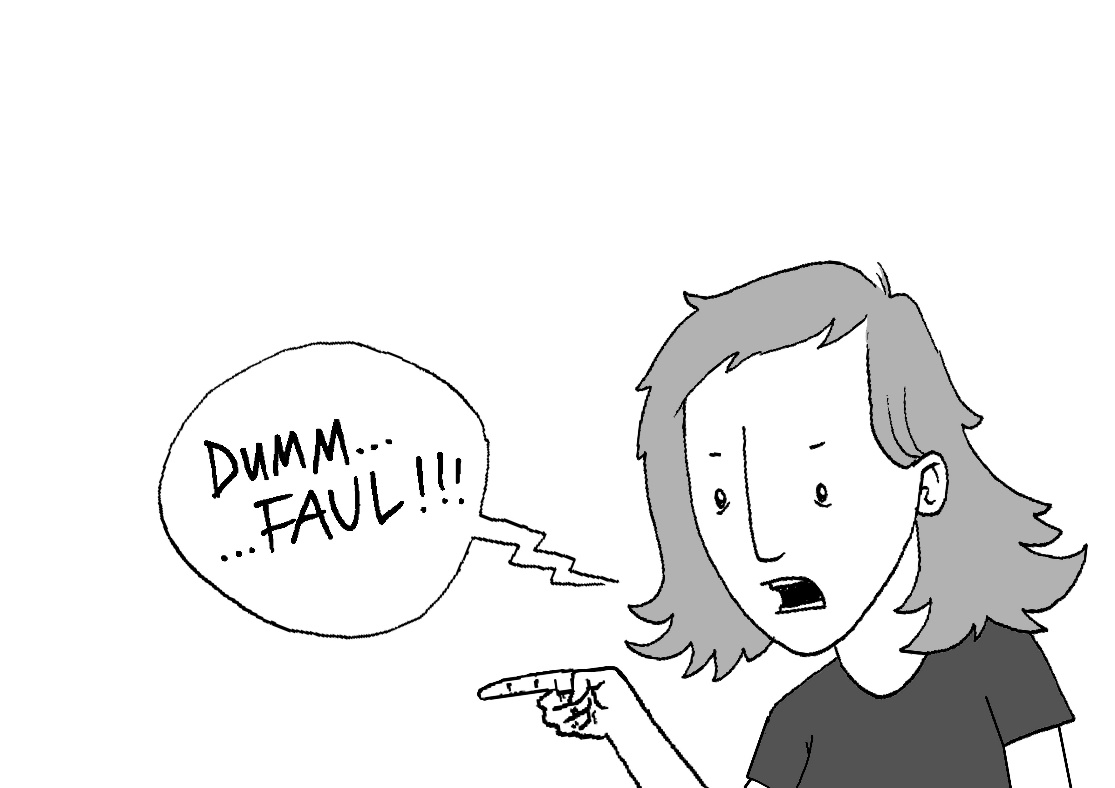


**Figure M1. Item 21 (target-parent: mother): “call the child dumb or lazy”.**


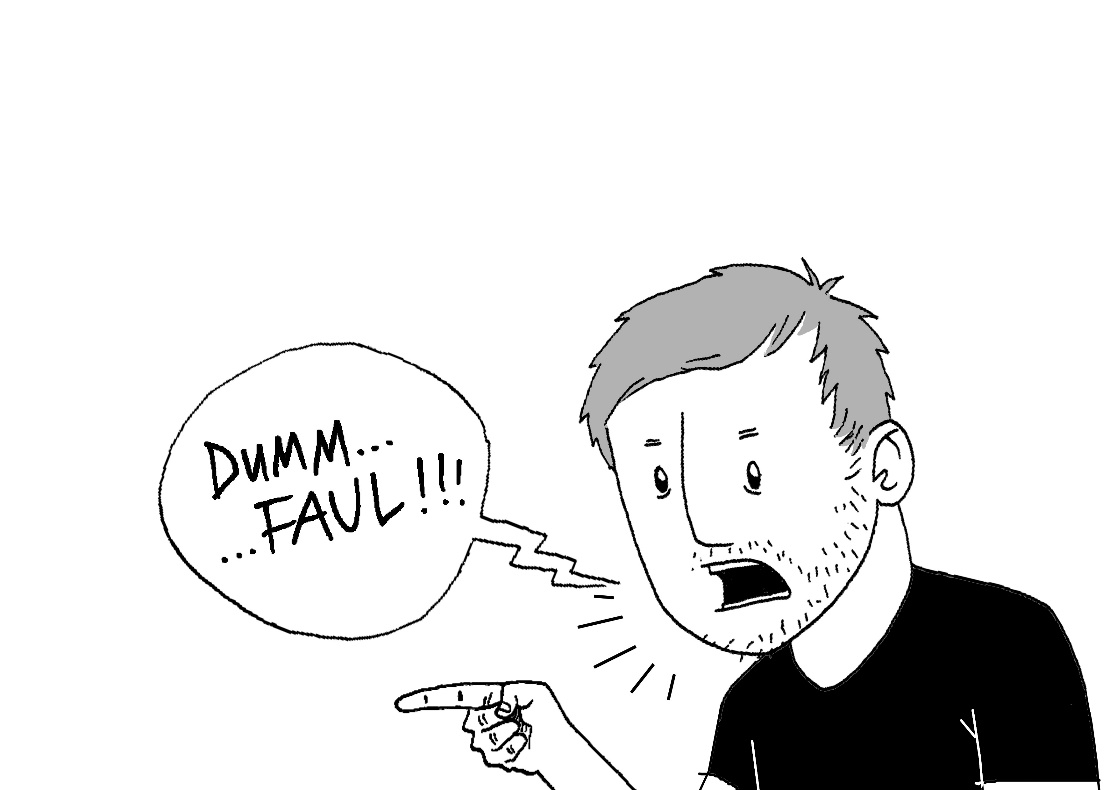


**Figure M2. Item 21 (target-parent: father): “call the child dumb or lazy”.**


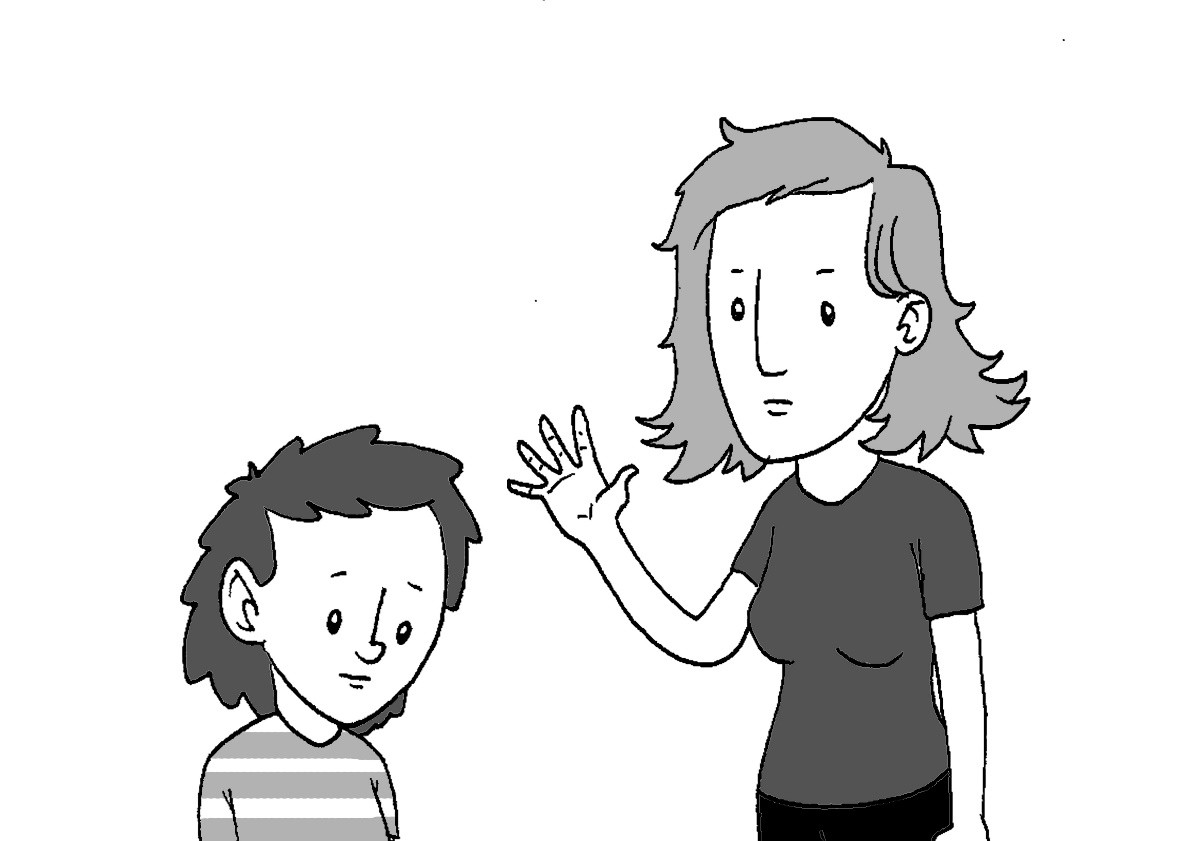


**Figure N1. Item 22 (target-parent: mother): “slap the child on the face”.**


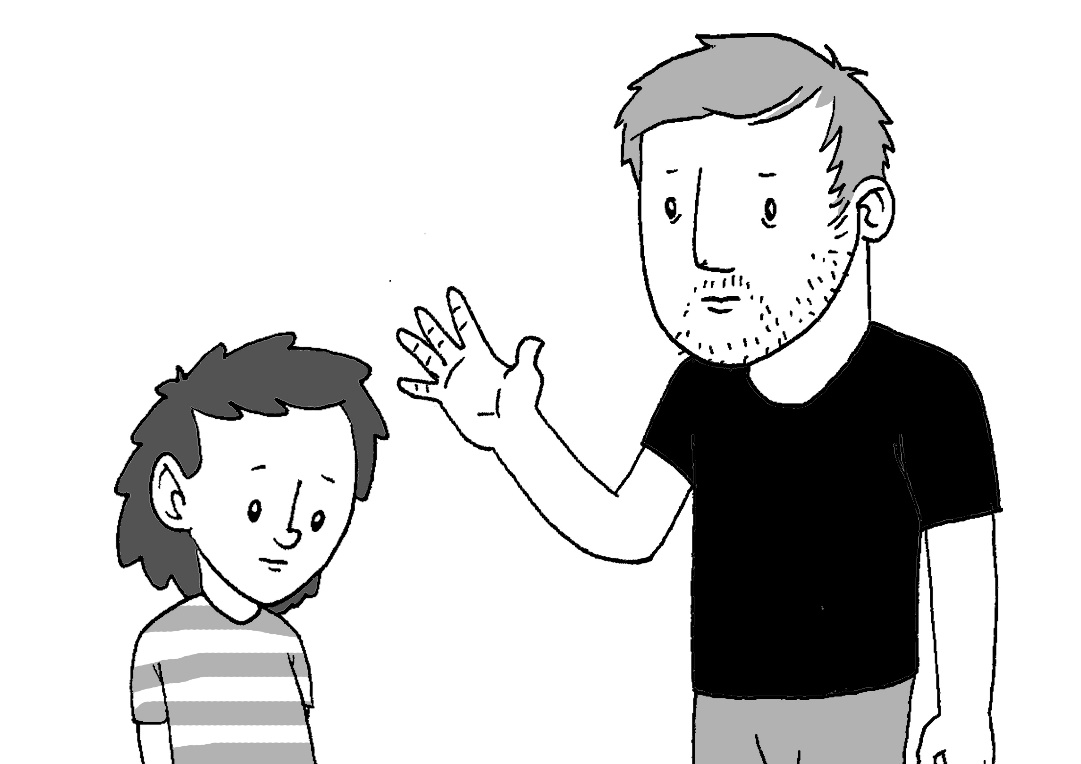


**Figure N2. Item 22 (target-parent: father): “slap the child on the face”.**


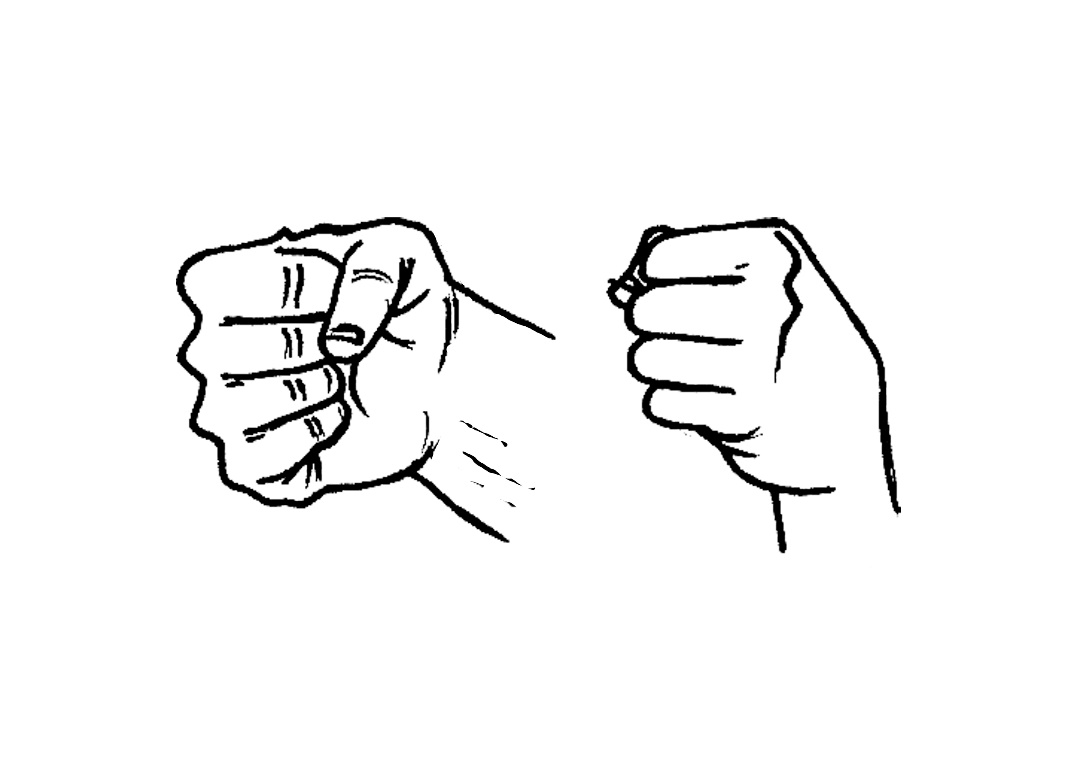


**Figure O. Items 7 and 11: “punch or kick the child” and “beat the child up”.**


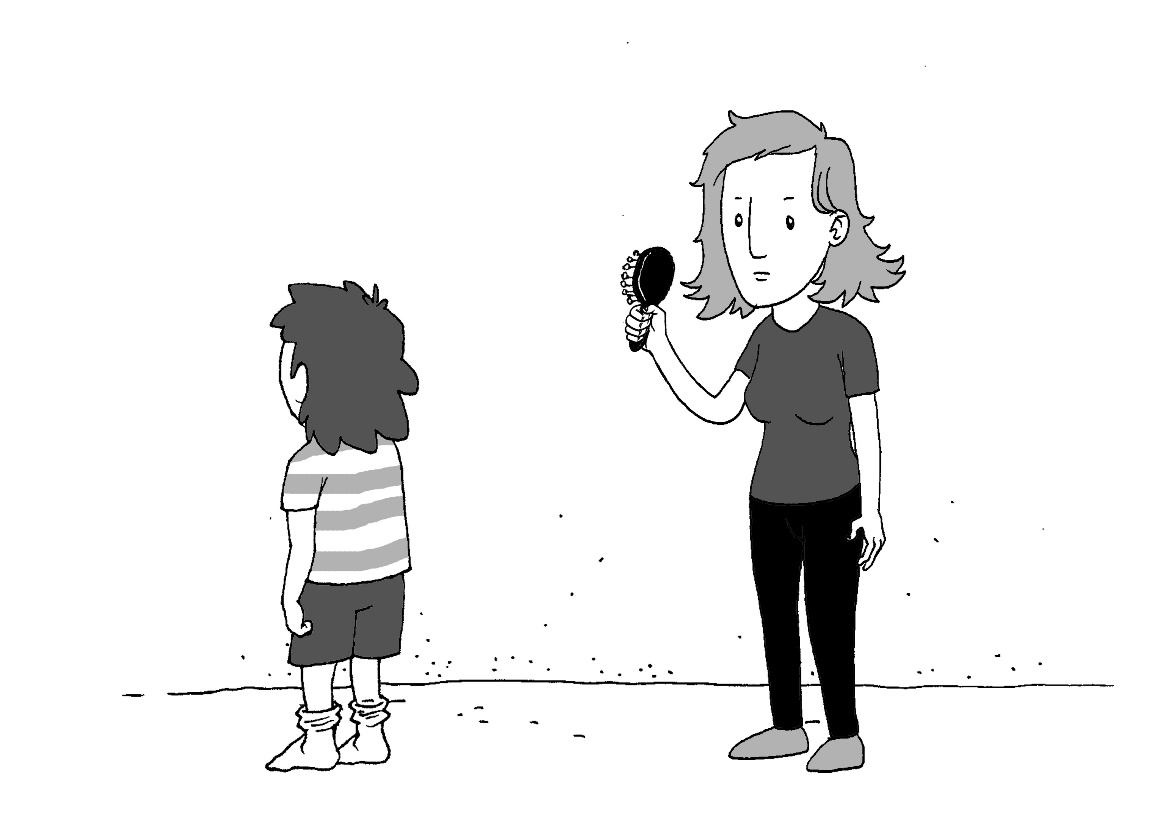


**Figure P1. Item 4 (target-parent: mother): “hit the child on the bottom with something hard”.**


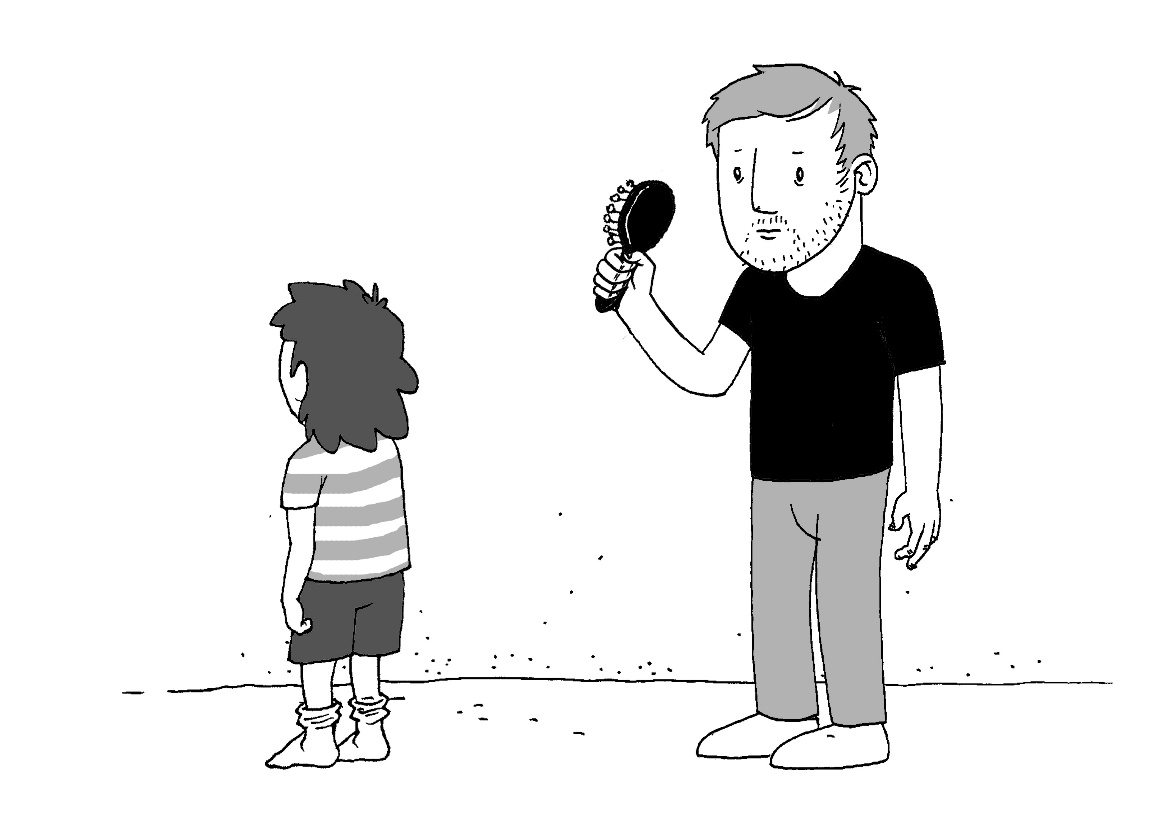


**Figure P2. Item 4 (target-parent: father): “hit the child on the bottom with something hard”.**


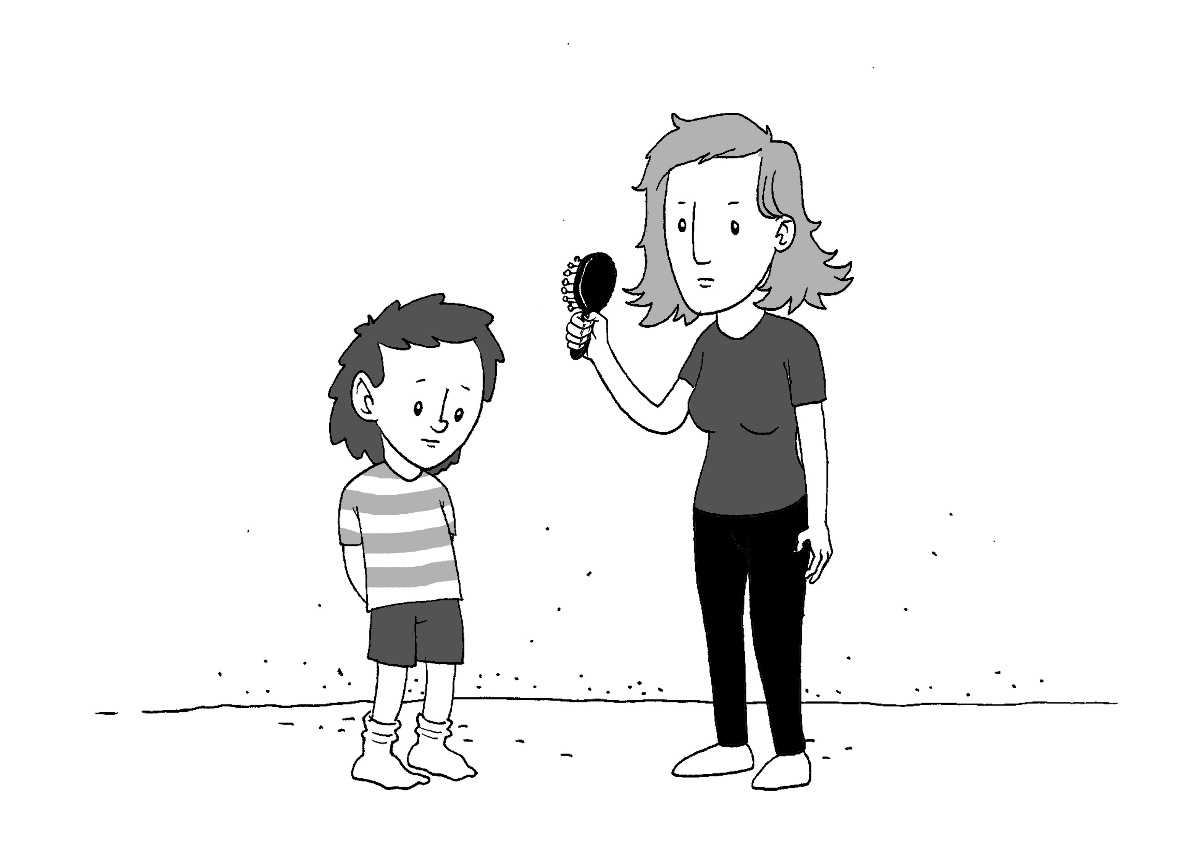


**Figure Q1. Item 15 (target-parent: mother): “hit the child with something hard”.**


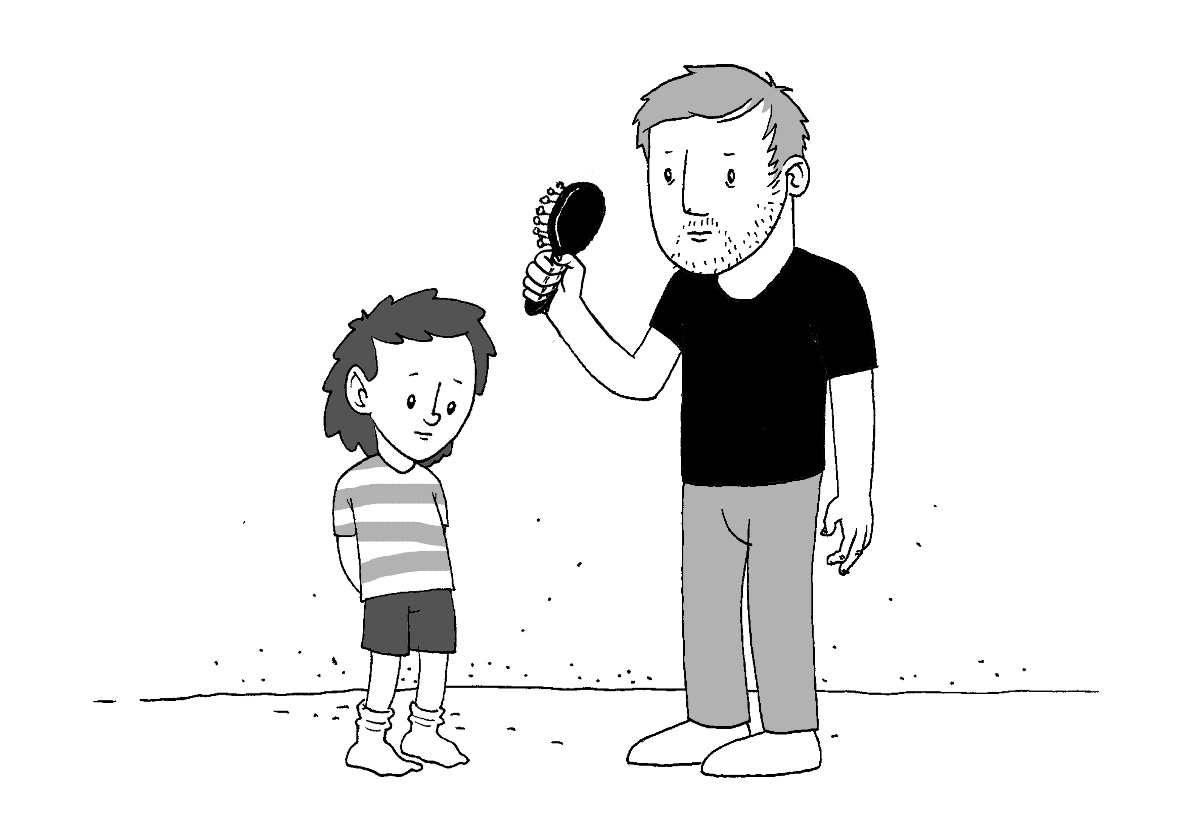


**Figure Q2. Item 15 (target-parent: father): “hit the child with something hard”.**


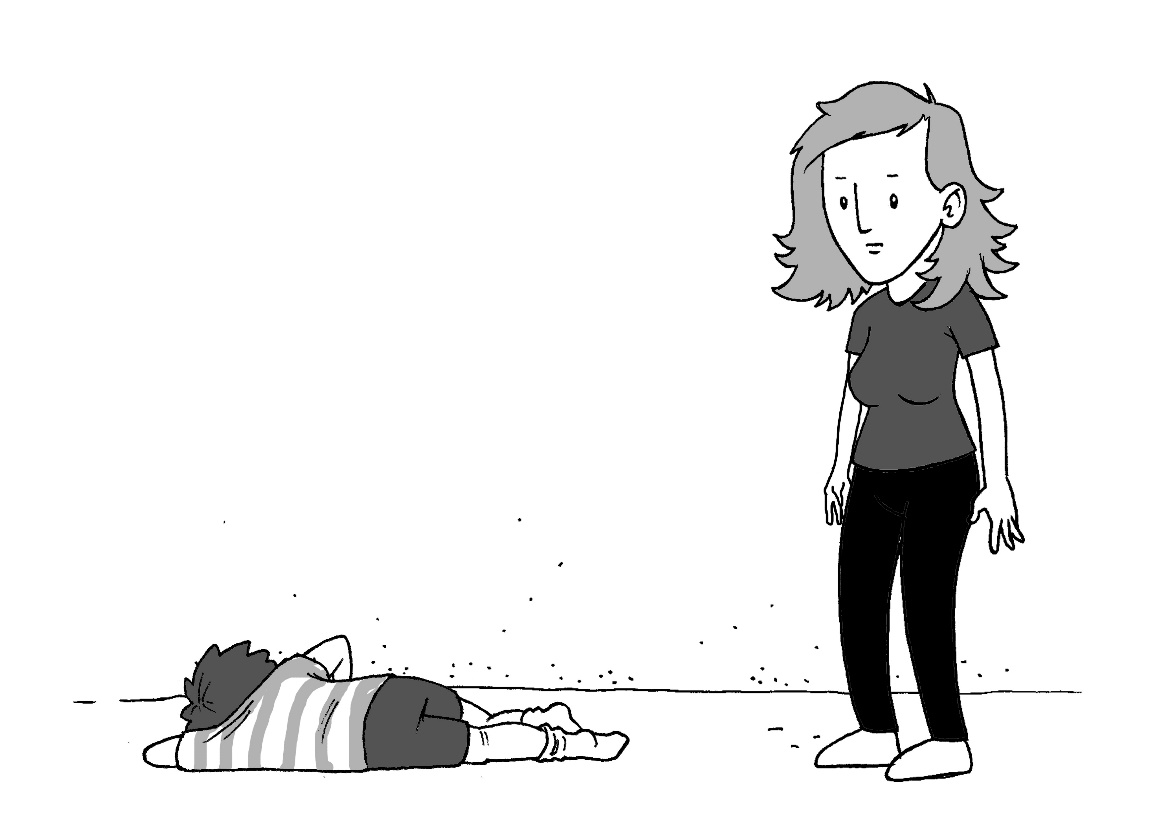


**Figure R1. Item 20 (target-parent: mother): “throw or knock the child down”.**


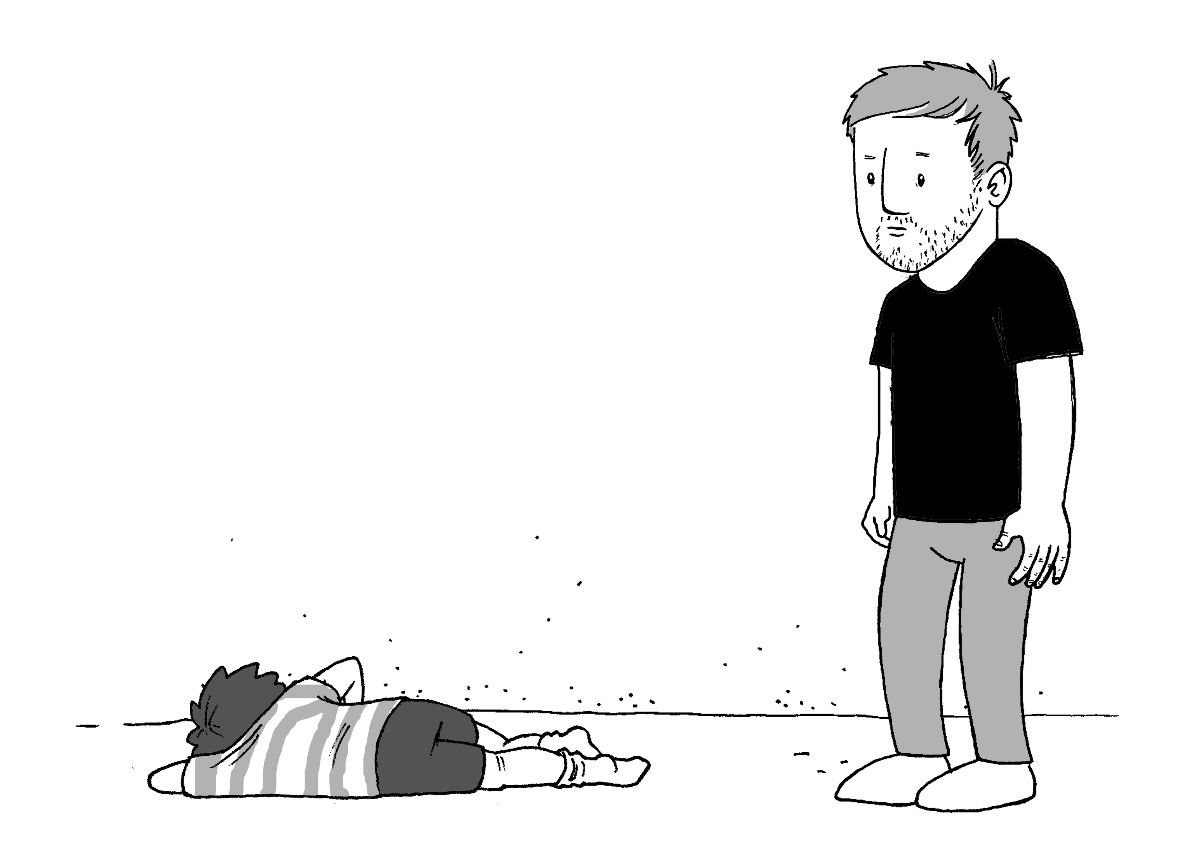


**Figure R2. Item 20 (target-parent: father): “throw or knock the child down”.**


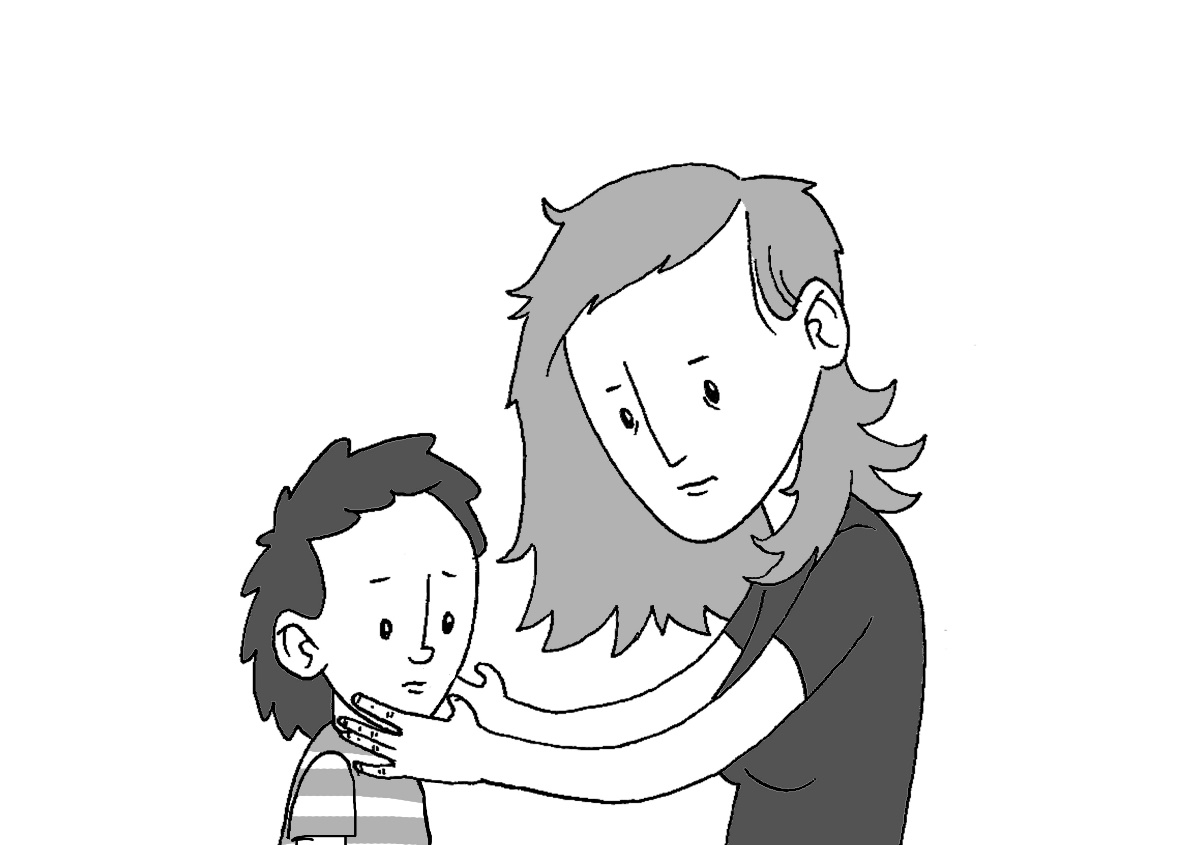


**Figure S1. Item 9 (target-parent: mother): “grab the child around the neck and choke him/ her”.**


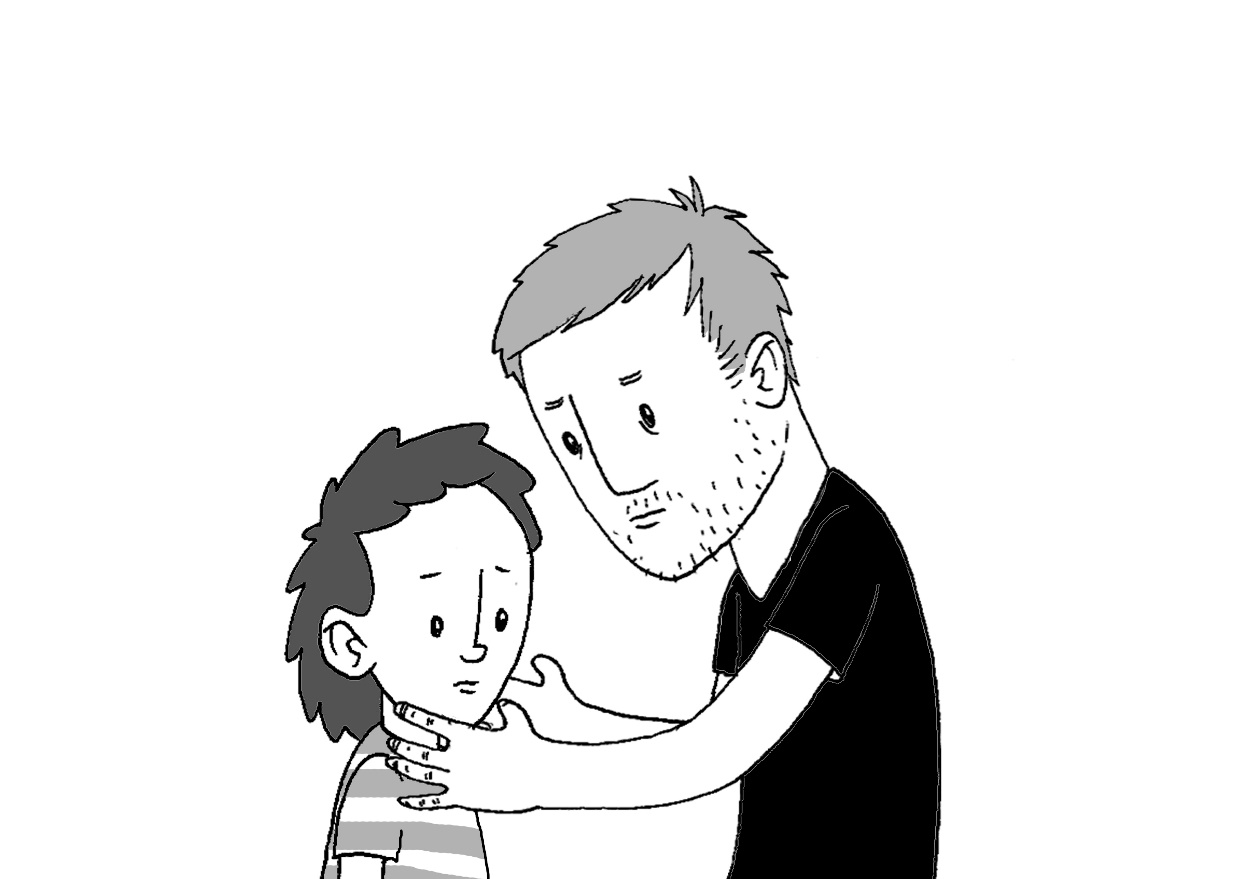


**Figure S2. Item 9 (target-parent: father): “grab the child around the neck and choke him/ her”.**


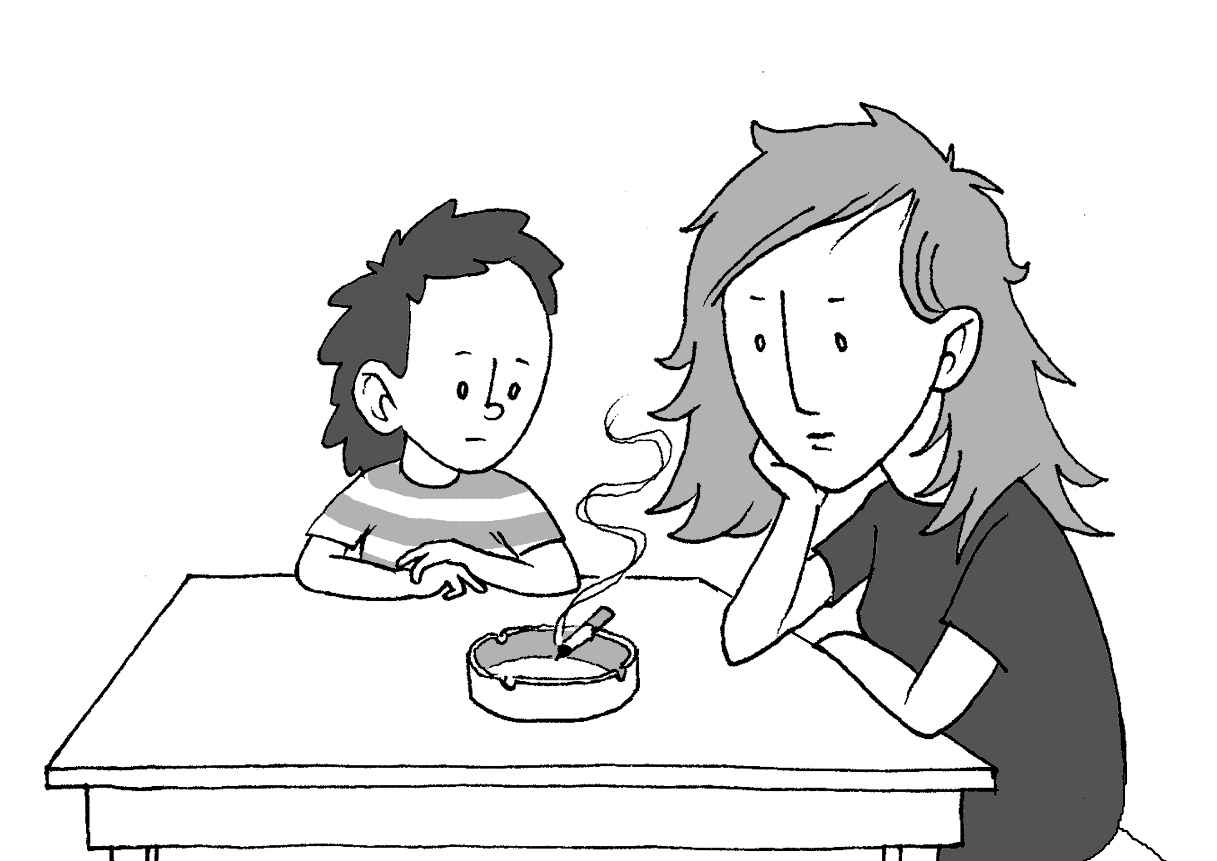


**Figure T1. Item 13 (target-parent: mother): “burn the child on purpose”.**


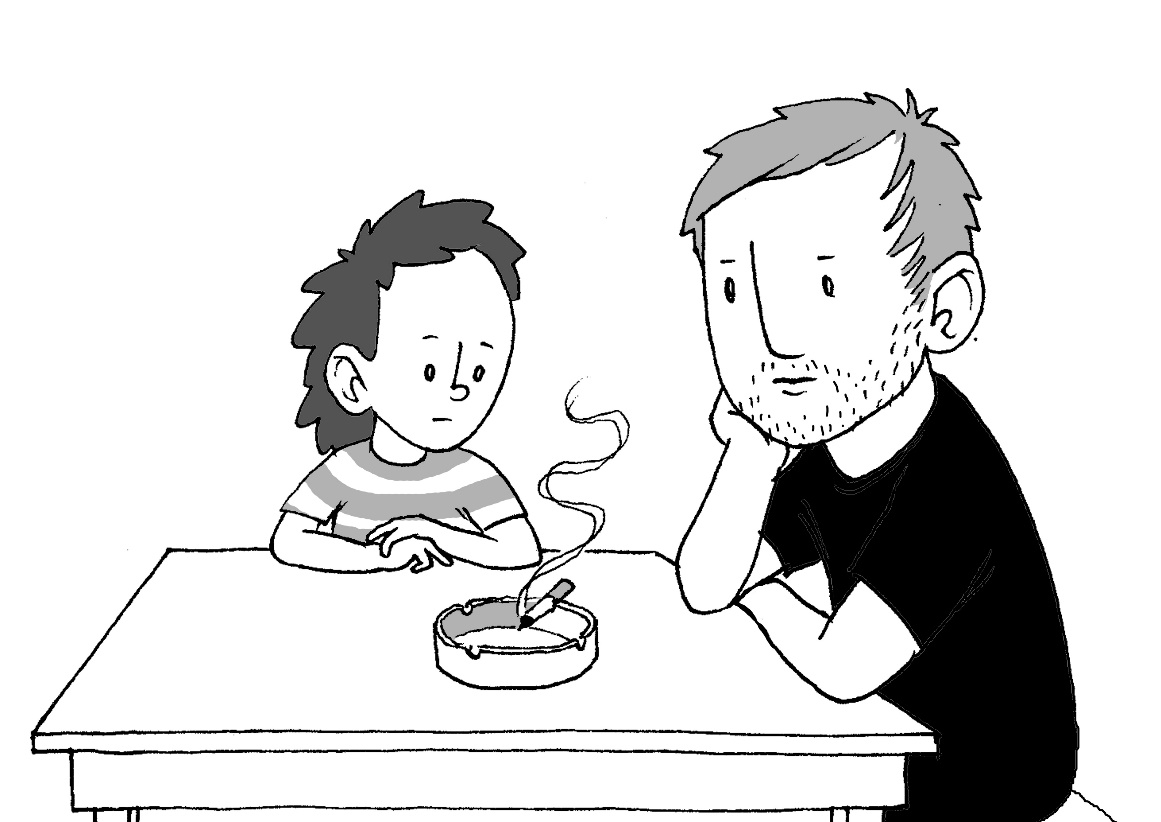


**Figure T2. Item 13 (target-parent: father): “burn the child on purpose”.**


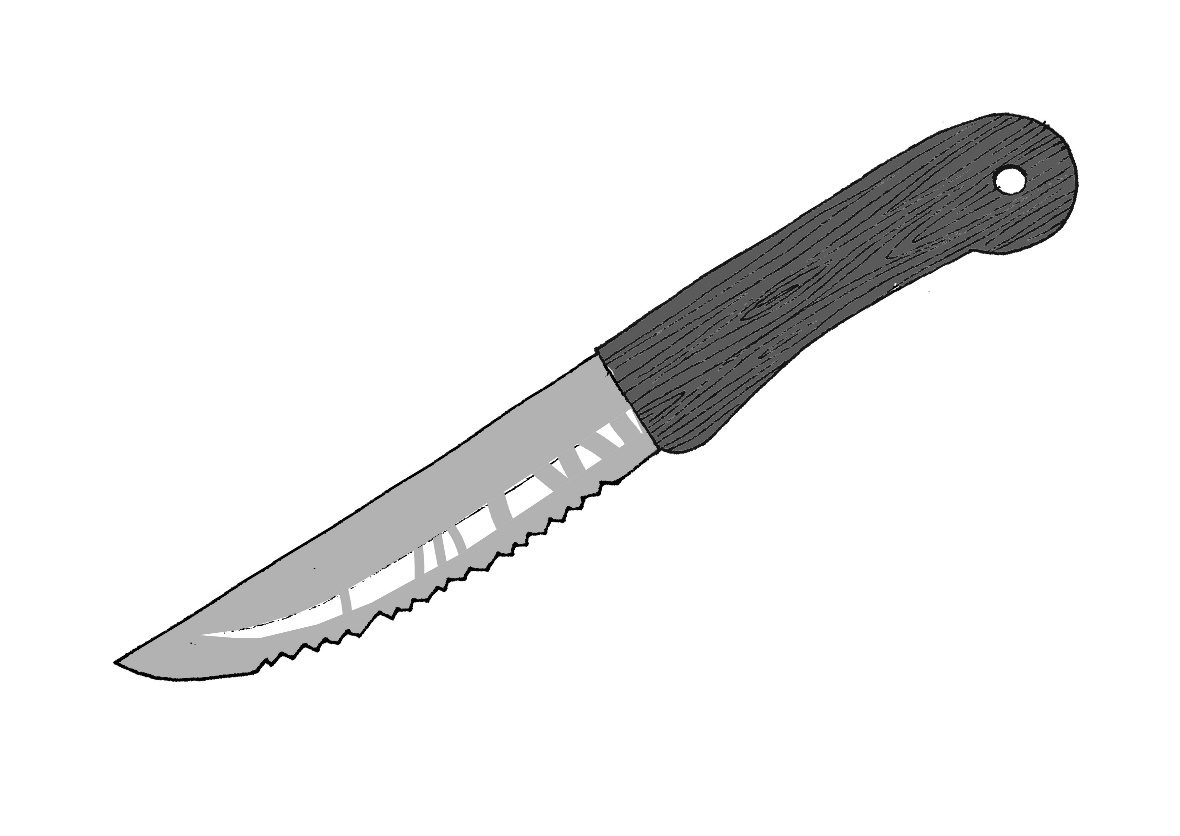


**Figure U. Item 19: “threaten the child with a knife or a gun”.**
